# Changes in Volatile Compounds and Sensory Properties of Chicken with *Armillaria mellea* During the Pressure-Cooking Process

**DOI:** 10.3390/foods14010083

**Published:** 2025-01-01

**Authors:** Xiaolan Dong, Chuntao Xia, Hongxiu Fan, Xu Zhang, Tong Sun, Zhiyu Wang, Tingting Liu

**Affiliations:** 1 College of Food Science and Engineering, Jilin Agricultural University, Changchun 130118, China; dxl20001202@126.com (X.D.); 15843642571@163.com (C.X.); fanhongxiu20071105@126.com (H.F.); suntong08051320@163.com (T.S.); 15145813791@163.com (Z.W.); 2 Scientific Research Base of Edible Mushroom Processing Technology Integration of Agriculture Ministry and Rural Affairs Ministry, Changchun 130118, China; 3 Engineering Research Center of Grain Deep-Processing and High-Efficiency Utilization of Jilin Province, Changchun 130118, China; 4 Jilin Province Product Quality Supervision and Inspection Institute, Changchun 130103, China; zhangxu@jlzjy.org

**Keywords:** chicken with *Armillaria mellea* prepared via pressure cooking, gas chromatography–mass spectrometry (GC-MS), gas chromatography–ion mobility spectrometry (GC-IMS), odor activity value (OAV), sensory properties

## Abstract

Chicken with *Armillaria mellea* prepared via pressure cooking is a traditional Chinese delicacy with great potential for food development. Optimizing its cooking time is crucial. In this study, chicken and *Armillaria mellea* were pressure-cooked for different amounts of time (20 min, 25 min, 30 min, 35 min, and 40 min). In total, 101 and 81 volatile compounds were identified by GC-MS and GC-IMS, respectively. The results showed that the content of volatile compounds was the highest at 40 min. Nonanal, decanal, (E,E)-2,4-nonadienal, (E,E)-2,4-decadienal, and 1-octen-3-ol were identified as the most critical aroma compounds at this time, which brought unique fat, oil, and mushroom aroma to chicken with *Armillaria mellea* during the pressure-cooking process. The optimal time was determined to be 35 min through sensory properties. In summary, the optimal cooking time for chicken with *Armillaria mellea* prepared via pressure cooking is 35–40 min. Our research results not only preliminarily determined the optimal conditions for industrial processing of the prepared dish of with *Armillaria mellea* prepared via pressure cooking, laying a foundation for the later industrial production of prepared dishes and international sales, but also stimulated innovative composite food development and promoted people’s exploration of the mechanism of heat treatment on composite food flavor and taste.

## 1. Introduction

As a precious traditional dish of Northeast China, chicken with *Armillaria mellea* is cooked by pressure and is made from chicken and wild Armillaria mellea from Changbai Mountain. With its long history, regional significance, and unique flavor, it is deeply loved by consumers and has been passed down to this day. As Chinese food is becoming increasingly popular internationally, the pre-prepared dish of chicken with *Armillaria mellea* prepared via pressure cooking has become popular, allowing more people taste this delicacy. Somebody can enjoy this delicious dish by heating it wherever.

*Armillaria mellea* is widely available, especially in Northeast China. It is not only edible but also has high medicinal value [1,2]. However, current research on *Armillaria mellea* focuses on its medicinal uses, such as fighting diabetes [3,4] and delaying depression [5]. Research on their flavor and taste is relatively rare. The most famous thing about chicken with *Armillaria mellea* prepared via pressure cooking is its smell. In order to spread this unique flavor, many food products with this flavor are produced, like chicken and *Armillaria mellea* stew instant noodles and chicken and *Armillaria mellea* stewed pre-prepared dishes. However, the source of its unique aroma has not been explored.

Current research on cooked chicken and mushroom flavors found that the key flavor compounds in cooked chicken include hexanal, nonanal, trans-2-decenal, and 1-octen-3-ol, contributing to fruity, green, mushroom-like, and meaty aromas [6]. Similarly, mushroom-like flavors in edible fungi are attributed to compounds such as 1-octen-3-ol and 2-octen-1-ol, which vary in proportion based on mushroom species [7]. On the other hand, flavors can also be affected by food-processing methods. For example, the volatile compounds in chicken soup tend to stabilize after approximately 2 h of simmering [8]. Moreover, it is found that prolonging the simmering time positively impacts flavor enhancement [9]. Similarly, the smoking durations were shown to affect the intensity of smoked flavor in chicken thighs, with the sample smoked for 4 min being most preferred by consumers [10]. Stewing in *Agaricus bisporus* and *Pleurotus ostreatus* is noted for its increased aromatic complexity compared to baking [11].

The dynamic alterations In volatile”comp’unds and sensory properties throughout the pressure-cooking process of chicken and *Armillaria mellea* remain unexplored. This knowledge gap stems from the phenomenon whereby the disruption of cell membranes and cell walls of mushrooms during heating results in the release of chemicals during cooking, amplifying individual substances’ content [12]. Moreover, higher temperature and pressure, coupled with prolonged braising durations, can induce the oxidation of fatty acids, thus facilitating the degradation of lipids and instigating Maillard reactions in meat, resulting in a broader spectrum of volatile compounds [13,14]. However, the complexity of these reactions during processing poses further challenges for the instrumental detection of the generated volatile compounds.

Consequently, there is significant potential for exploring the source of chicken with *Armillaria mellea* during the pressure-cooking process, its unique aroma, research into the dynamic changes in volatile compounds during the pressure-cooking processing, and its sensory properties. In this study, we used *Armillaria mellea* and chicken as primary ingredients and subjected them to pressure cooking to facilitate interaction. By combining GC-MS, GC-IMS techniques, texture analysis, and E-sensory properties systems (Electronic-nose (E-nose), Electronic-tongue (E-tongue), and sensory properties), we aim to achieve three objectives: (1) investigate the changes in volatile compounds and sensory properties of chicken with *Armillaria mellea* during the pressure-cooking process; (2) characterize its aroma characteristics and identify the most contributing aroma compounds; and (3) Determine the optimal pressure-cooking time. Internationally, the prepared-food industry is growing rapidly, making cooking easier and quicker and helping consumers take a break from the hassle of meal preparation. As a result, this research endeavor will provide theoretical insights into producing high-quality pre-made dishes featuring chicken with *Armillaria mellea* prepared via pressure cooking and offer consumers healthier and more delectable convenience foods. At the same time, it provides a theoretical basis for local specialty food to go to the international stage. Furthermore, it lays a foundation for the long-term development of food factories, such as food products with their characteristic flavors (e.g., pre-made dishes and other convenience food).

## 2. Materials and Methods

### 2.1. Sample Preparation and Processing

Wild *Armillaria mellea* was harvested from Changbaishan Mountain (Baishan, China), and drumsticks and salt were obtained from Charoen Pokphand Group (Changchun, China). In order to obtain the best chicken flavor and taste, we choose to use only the drumsticks of chickens. The chicken and *Armillaria mellea* were meticulously cleaned. According to the results of the previous single-factor test, the best ratio of chicken and *Armillaria mellea* was determined to be 1:11. Each group of samples was prepared according to this ratio, and each group weighed 300 g. Each group was treated with 600 mL of deionized water and subjected to a controlled simmering process in a pressure cooker, under an average pressure of 70 kPa. This established the control group, S20, S25, S30, S35, and S40. Pressure-cooking durations were meticulously regulated at 20 min, 25 min, 30 min, 35 min, and 40 min, respectively, and there were three parallel experiments in each time period.

### 2.2. E-Nose Analysis

E-nose analysis was conducted using the PEN 3 electronic nose model (Winmuster Airsense Analytics Inc., Schwerin, Germany). Prior to analysis, 5 g of suspension for each sample was accurately weighed, placed in a 40 mL injection bottle, and then incubated at 25 °C for 2 h to reach the headspace equilibrium. Sampling parameters for the E-nose were configured as follows: test time—60 s; cleaning time—90 s; internal sensor flow rate—300 mL/min; and sample injection flow rate—300 mL/min. Equilibration of all samples was achieved within approximately 55–58 s. The signal-processing system digitized analog signals, and computer analysis obtained output results. In combination with the principal component analysis (PCA) algorithm, data collected in high-dimensional space within the machine were reduced to low-dimensional space [15] to efficiently identify the types of odors present in the food samples.

### 2.3. E-Tongue Analysis

The SA402B Electronic tongue (E-tongue) analysis system produced by the INSENT company (Intelligent Sensor Technology Co. Ltd., Fukuoka, Japan) was used to efficiently identify the types of taste present in food samples. The detection sensors used in this analysis include AAE (for umami), CTO (for salty), CAO (for sour), COO (for bitter and aftertaste-B), and AE1 (for astringency and aftertaste-A), along with two reference electrodes. An approach based [16] on was slightly modified: cleaning solution for 90 s, 120 s, and 120 s; conditioning solution for 30 s; and detection time set to 30 s. In order to obtain the pure-flavor solution, the samples were cooled to 25 °C, homogenized, and beaten using an SLS-60-70 homogenizer (Shanghai, China) at 43,000 rpm for 2 min to obtain the corresponding suspensions. Then, 100 mL of the suspension of each sample was centrifuged at 4 °C and 2164× *g* for 20 min, and the filtered upper clear liquid was used for E-tongue analysis.

### 2.4. Detection of the Volatile Compounds Using GC-MS

The detection and analysis of volatile compounds were conducted using a headspace solid-phase micro-extraction (HS-SPME) technique coupled with gas chromatography–mass spectrometry (GC-MS). Parameter settings for the GC-MS and SPME measurements were modified based on [17]. Each sample suspension, weighing 4 g, was dispensed into an 8 mL SPME headspace vial, mixed with 1 μL of 2-methyl-3-heptanone (1 μg/μL) as internal standard, and then sealed with a polytetrachloroethylene spacer. Incubate at 20 °C for 30 min to reach headspace equilibrium prior to injection, a 50/30 μm DVB/CAR/PDMS composite extraction head was inserted into the headspace vial and allowed to adsorb for 40 min at 60 °C, the entire SPME process was 70 min before injection into GC-MS. Subsequently, the fiber head within the extracted composite extraction head was expelled and desorbed for 5 min at 250 °C before reinserting into the GC inlet. The HP-INNOWax highly polarized column (30 m × 0.25 mm, 0.25 mm) was used for the separation. The column temperature was initially maintained at 40 °C for 3 min, then ramped up to 80 °C at a rate of 5 °C/min and held for an additional 3 min, followed by an increase to 230 °C at a rate of 10 °C/min, and finally held at 230 °C for 5 min. High-purity helium (>99.999%) was utilized as the carrier gas, flowing at a 1.0 mL/min rate. Mass spectrometry was conducted in electron ionization (EI) mode at 70 eV, with the ion-source and transmission-line temperatures set at 240 °C and 230 °C, respectively. The mass range that was scanned ranged from 40 to 450 *m*/*z*.

### 2.5. Characterization and Quantification of the Volatile Compounds

Under the same chromatographic conditions, the retention index (RI) of the detected volatile compounds was calculated from the retention time of n-alkanes (C5–C30) by linear interpolation; n-ketones (C4–C9) were purchased from Sinopharm Chemical Reagent Co., Ltd., Shanghai, China; and n-alkanes (C7–C30) were purchased from Anpu Experimental Technology Co., Ltd., Shanghai, China, and compared with NIST05 and Wiley07 databases, where the absolute values differing by 50 or less were determined to be the same compound [18].

Quantitative analysis of volatile compounds was performed using internal standard methods. Peak areas were integrated based on the total ion chromatogram of volatile compounds. The relative contents of volatile metabolites were calculated by comparing the peak area with the internal standard.

Odor activity values (OAVs) were utilized to assess the contribution of each volatile compound to the overall aroma, calculated as the ratio of the quantitative analysis result of a single volatile compound (*Ci*) to the odor perception threshold in water (*T*) [19]. The calculation formulae are as follows:

(1) $$RI=100\times \left(\frac{t_{x}-t_{n}}{t_{n+1}-t_{n}}+n\right)$$

where *t_x_*, *t_n_*, and *t*_*n*+1_ are the retention times of the volatile compounds to be measured; and n-alkanes have *n* carbon atoms, while *n*+1 carbon atom n-alkanes, for which *t_x_*<*t_n_*<*t*_*n*+1_;

(2) $$C_{i}=\frac{A_{i}}{A_{S}\times m_{i}}\times m_{s}$$

where *C_i_* is the concentration of the volatile compounds (μg/kg); *A_i_* and *A_s_* are the peak areas of volatile compounds and that of the internal standard compounds, respectively; *m_i_* is the mass of the samples (g); and *ms* is the mass of the internal standard compounds (mg).

(3) $$OAV=\frac{C_{i}}{T}$$

where *C_i_* is the concentration of the volatile compounds (μg/kg), and *T* is the organoleptic threshold of the volatile compound.

### 2.6. Detection of Volatile Compounds Using GC-IMS

An approach based on [20] was slightly modified to use HS-GC-IMS (G.A.S. Gesellschaft für Analytische Sensorsysteme mbH, Dortmund, Germany). A 4 g sample was placed in a 20 mL headspace container and incubated at 100 °C for 30 min, with a speed of 500 rpm. Subsequently, 500 µL of headspace was automatically injected into an MXT-WAX capillary column (30 m × 0.53 mm, 1.0 μm) for chromatographic separation using a non-split injection when the column temperature reached 60 °C. High-purity nitrogen (purity ≥ 99.999%) was used as the carrier gas, and the injection port was kept at 80 °C. Initially, a 2.0 mL/min flow rate was maintained for 2 min, followed by a linear increase to 10.0 mL/min over 8 min, and then to 100.0 mL/min over 10 min, maintaining this flow rate for another 20 min. The overall run time for chromatography was 40 min. The n-alkanes (C4–C9) were used as an external reference for calculating the retention index (RI), and n-ketones (C4–C9) were purchased from Sinopharm Chemical Reagent Co., Ltd., Shanghai, China. The volatile metabolites were separated and identified based on the different migration rates and using the retention index (RI) and drift time (Dt) by searching the National Institute of Standards and Technology (NIST) library and the IMS database from G.A.S. (Dortmund, Germany). Volatile metabolite content was presented as signal peak area. Two-dimensional top views and volatile metabolite fingerprints were generated using two plugins (Reporter and Gallery Plot) in the GC-IMS system [21].

### 2.7. Texture Analysis

The samples’ hardness, chewiness, and gumminess indexes were detected using Texture Index software (no version, Stable Microsystems Ltd., Surrey, UK). According to the research of Dong [22], samples of pressure-cooked chicken and *Armillaria mellea* were prepared as 1 cm × 1 cm × 1 cm squares, sliced parallel to the direction of chicken fibers. These samples were compressed using a cylindrical probe (p 0.5) on a test stand. For chicken, the test parameters were as follows: a cross-probe falling speed of 2.0 mm/s, a test speed of 1.0 mm/s, and a rising speed of 2.0 mm/s at the end of the test. A 3 s interval between the first and second presses was maintained, with a working compression ratio of 65% and a test height of 25 mm. For *Armillaria mellea*, the parameters were adjusted slightly: a cross-probe descending speed of 2.0 mm/s, a test speed of 1.0 mm/s, and a rising speed of 2.0 mm/s at the end of the test. A 5 s interval between the first and second pushes was applied, with a working compression ratio of 60% and a test height of 25 mm.

### 2.8. Sensory Properties

Twelve trained panelists (50% males and 50% females, aged 20 to 30) were used, and each panelist had received over 240 h of sensory evaluation training. The panelists were first trained in taste, including sour, sweet, bitter, salty, and umami, using 0.02 mL citric acid, 0.05 mL sucrose, 0.001 mL caffeine, 0.02 mL salt, and 0.01 mL monosodium glutamate [23]. The unhomogenized samples were analyzed across four dimensions: appearance, odor, taste, and flavor. Ratings were conducted on a 25-point scale, with scores ranging from 0 to 9 indicating unacceptable, from 10 to 14 indicating barely acceptable, from 15 to 20 indicating basically acceptable, and from 21 to 25 indicating completely acceptable. Samples were divided into twelve equal parts for each panelist, uniformly packaged, and randomly labeled with the numbers 1–5. Panelists were isolated from one another to ensure sample distribution homogeneity. During the 30 s tasting phase, panelists rinsed their mouths with water and consumed unsalted soda crackers to eliminate residual taste [24].

### 2.9. Subsection Statistical Analysis

Each experiment was performed in three replicates. Statistical analyses were conducted using the Statistical Package for the Social Sciences (SPSS, Chicago, IL, USA). Results were presented as mean ± SD. Differences between groups’ means were assessed using one-way ANOVA, with an LSD post hoc test performed to evaluate the statistical difference; different letters indicate significant differences (*p* < 0.05).

The Winmuster software was utilized to analyze PCA and E-nose data processing. E-tongue data processing was performed using the Taste analysis application. Line and bar charts depicting volatile compounds by GC-MS and radar fingerprints of E-nose aroma profiles were created using Origin 2021 (OriginLab, Northampton, MA, USA).

## 3. Results and Discussion

### 3.1. Analysis of the Aroma Profile of Chicken with Armillaria mellea During the Pressure-Cooking Process by E-Nose

**Figure 1:**
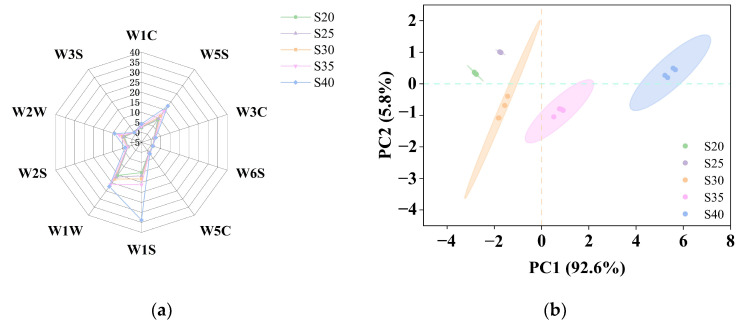
E-nose analysis of chicken with *Armillaria mellea* prepared via pressure cooking for different pressure-cooking times. Radar chart (**a**) and principal component analysis (**b**) of the E-nose data of chicken with *Armillaria mellea* prepared via pressure cooking treated with different pressure-cooking times. E-nose sensors include W1C (sensitive to aromatic constituents, benzene), W5S (highly sensitive to nitrogen oxides), W3C (sensitive to ammonia), W6S (sensitive to hydrides), W5C (sensitive to olefin, short-chain aromatic compounds), W1S (sensitive to methane), W1W (sensitive to sulfides), W2S (sensitive to alcohols, aldehydes, and ketones), W2W (sensitive to organic sulfides), and W3S (sensitive to long-chain alkanes).

The E-nose, a biomimetic instrument, can exhibit certain variations in the detection of flavor compounds [25]. The changes indicated the overall trend of flavor development in chicken with *Armillaria mellea* during the pressure-cooking process. During the pressure cooking process, new volatile compounds are generated following a Maillard reaction, derived from *Armillaria mellea* and chicken, thus producing a distinct flavor [26]. As shown in Figure 1a, the intensity of the volatile compounds in the *Armillaria mellea* is influenced by the lengthening of the pressure-cooking period. Notably, sensors W1C (sensitive to aromatic constituents, benzene), W3C (sensitive to ammonia), W6S (sensitive to hydrides), W5C (sensitive to olefin, short-chain aromatic compounds), and W3S (sensitive to long-chain alkanes) exhibited minimal signal intensities. The differences between the groups were slight and had no significant impact on the overall flavor analysis. There is no significant change in the content, suggesting a lower content of various aromatic chemicals, ammonia compounds, hydrogen compounds, hydrocarbons, and methane during the pressure-cooking process. While sensors W2S (sensitive to alcohols, aldehydes, and ketones) and W2W (sensitive to organic sulfides) showed slight increases in response values, suggesting a marginal enhancement in taste compounds, such as ethanol, organosulfides, pyrazines, terpenes, etc., during pressure cooking. Moreover, the higher signal intensity and significant increase (*p* < 0.05) in response values for sensors W1S (sensitive to methane), W1W (sensitive to sulfides), and W5S (highly sensitive to nitrogen oxides) indicated the presence of numerous methyl groups in chicken with *Armillaria mellea* prepared via pressure cooking, which is sensitive to sulfides, pyrazines, and nitrogen oxides. Therefore, extending the pressure-cooking time intensified the presence of these flavor substances.

To gain a comprehensive understanding of the influence of different heating times on the flavors of chicken with *Armillaria mellea* prepared via pressure cooking, further PCA was conducted based on the E-nose finding. The contribution rates of principal component 1 (PC1) and principal component 2 (PC2) were 92.6% and 5.8%, respectively, with a cumulative contribution rate of 96.8% (Figure 1b). This high cumulative contribution rate (>85.0%) indicates that PC1 and PC2 effectively capture most of the sample information. The tight clustering of data points for each sample group, without overlap, suggests significant variation in volatile flavor compounds among the five samples (*p* < 0.05), accurately distinguishable by the E-nose. On the PC1 axis, samples S35 and S40 are positively distributed, and samples S20, S25, and S30 are negatively distributed. On the PC2 axis, samples S20, S25, and S40 are positively distributed, and samples S30 and S35 are negatively distributed, and samples overlap. These results indicate an enhancement in flavor substance intensity in chicken with *Armillaria mellea* prepared via pressure cooking with increasing pressure-cooking times. However, further analyses such as GC-MS and GC-IMS are warranted to validate this conclusion.

### 3.2. Analysis of the Taste Changes in Chicken with Armillaria mellea During the Pressure-Cooking Process by E-Tongue

**Figure 2:**
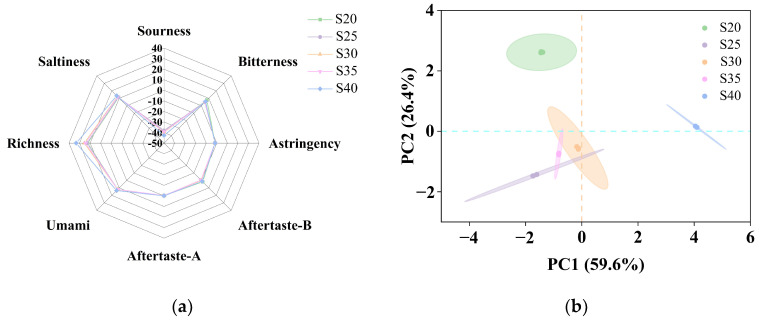
E-tongue analysis of chicken with *Armillaria mellea* prepared via pressure cooking for different pressure-cooking times. Radar chart (**a**) and principal component analysis (**b**) of the E-tongue data of chicken with *Armillaria mellea* that was pressure-cooked for different pressure-cooking times.

While the chemical composition of food is diverse, taste perception primarily comprises five basic sensations: salty, sour, sweet, umami, and bitter. In order to mitigate the subjectivity inherent in sensory properties [13], we examined changes in taste-related compounds in samples subjected to high-pressure pressure cooking under different time conditions in different groups, using an E-tongue. The radar map (Figure 2a) illustrated that all flavor indices except sourness were discernible, with values for all other taste components exceeding zero. Consequently, the predominant flavor profile of chicken with *Armillaria mellea* prepared via pressure cooking consists primarily of umami, salty, bitter, and astringent notes. Notably, umami and richness (an aftertaste of the umami) emerges as the most prominent flavor in all samples, closely followed by salty taste, indicating that umami predominates throughout the pressure-cooking process, increasing its abundance with prolonged, high pressure-cooking time. This phenomenon can be mainly attributed to the thermal treatment of proteins, resulting in elevated levels of free amino acids and short peptides, imparting an umami taste [12,27]. With the salt content held constant across all samples, no discernible variation in salty flavor was observed. At 20 min of pressure cooking, a hint of bitterness may arise due to the exposure of certain hydrophobic groups during the pressure-cooking process. However, as the pressure-cooking time extends, the bitterness gradually diminishes. This attenuation of bitterness could be attributed to the masking effect of meat flavor on bitter compounds present in *Armillaria mellea* during pressure cooking [28]. Meanwhile, the values for astringency, aftertaste-A, and aftertaste-B of chicken with *Armillaria mellea* prepared via pressure cooking were either lower or slightly higher than the blank, indicating minimal presence of astringency, aftertaste-A, or aftertaste-B bitterness in chicken with *Armillaria mellea* prepared via pressure cooking.

The taste-reaction values were subjected to PCA to observe further the effect of simmering time on the flavor of chicken with *Armillaria mellea* prepared via pressure cooking. As depicted in Figure 2b, PC1 and PC2 contributed 59.6% and 26.4%, respectively, cumulatively accounting for 86% of the variance. The result suggests that these principal compounds effectively capture most taste characteristics across the five sample groups. On the PC1 axis, only sample S40 is positively distributed, and samples S20, S25, S35, and S30 are negatively distributed. On the PC2 axis, samples S20 and S40 are positively distributed, and samples S25, S30, and S35 are negatively distributed.

This shows that chicken with *Armillaria mellea*, during the pressure-cooking process, predominantly exhibits a robust umami taste. Although a slight bitterness may manifest initially, prolonged pressure cooking gradually masks the bitterness present in *Armillaria mellea*. Given the absence of spoilage, sour-taste development was not observed. This suggests that altering the pressure-cooking time could effectively enhance the food’s release of diverse taste compounds, resulting in a more delightful flavor profile. Additionally, complex cooking techniques can mitigate inherent flavor defects in the food, enhancing the overall sensory experience.

### 3.3. Analysis of the Changes in the Content of Volatile Compounds in Chicken with Armillaria mellea During the Pressure-Cooking Process by GC-MS

**Figure 3:**
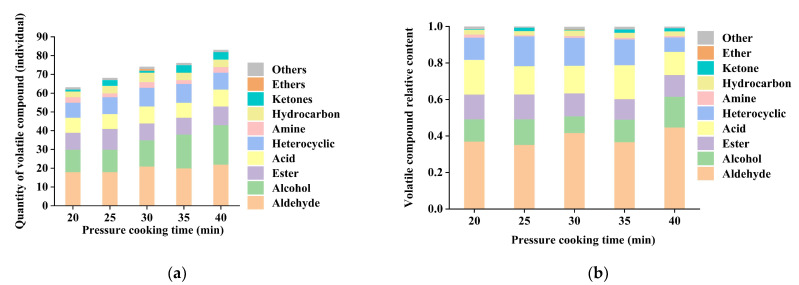
Analysis of volatile compounds, using SPME-GC-MS, in chicken with *Armillaria mellea* prepared via pressure cooking. (**a**) Number of volatile compounds detected among different groups. (**b**) Relative content of volatile flavor compounds across each group.

**Figure 4:**
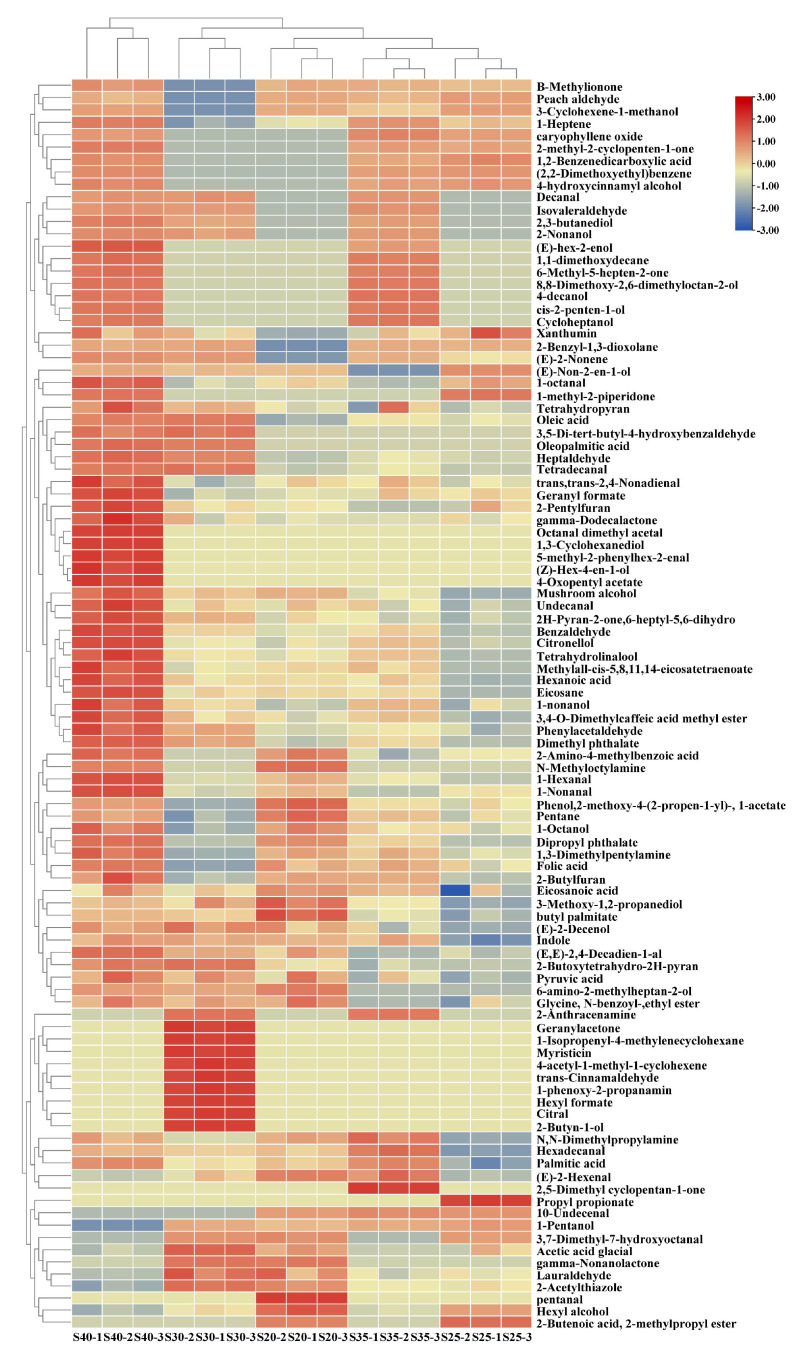
Heat map illustrating clustering results of volatile compound concentrations in chicken with *Armillaria mellea* prepared via pressure cooking for various pressure-cooking times. The color of the squares in the chart indicates the number of volatile compounds in chicken with *Armillaria mellea* prepared via pressure cooking. The color ranges from blue to red, with the relative content increasing from low (blue) to high (red). A darker blue color signifies a lower level of compounds, while a darker red color signifies a higher level of compounds.

To better understand the changes in the flavor compounds of the chicken with *Armillaria mellea* during the pressure-cooking process, GC-MS was used to analyze the volatile compounds in the samples with different pressure-cooking times. As shown in Supplementary Table S1, a total of 101 volatile compounds were detected by GC-MS in the five samples, which were categorized into 10 groups: 28 aldehydes, 23 alcohols, 13 esters, 11 heterocyclic compounds, 9 acids, 6 ketones, 5 hydrocarbons, 4 amines, 1 ether, and 1 other (Figure 3b). Notably, the number of volatile compounds fluctuated across samples with different pressure-cooking treatments: 20 min has 63 types, 25 min has 68 types, 30 min has 74 types, 35 min has 76 types, and 40 min has 83 types (as depicted in Figure 3a). Furthermore, to delve deeper into the changes in volatile compounds during the pressure-cooking process, a heatmap of the five samples was constructed, illustrating a discernible divergence in volatile flavor compounds (*p* < 0.05) (refer to Figure 4).

**Figure 5:**
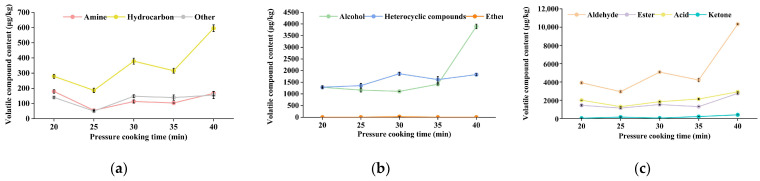
Changes in volatile compounds among different groups. Data are presented as mean ± SD. (**a**) The content of amines, terpenoids, and others in chicken with *Armillaria mellea* prepared via pressure cooking for various pressure-cooking times. (**b**) The content of alcohols, heterocycles, and ethers in chicken with *Armillaria mellea* prepared via pressure cooking treated for various pressure-cooking times. (**c**) The content of aldehydes, esters, acids, and ketones in chicken with *Armillaria mellea* prepared via pressure cooking for various pressure-cooking times.

S40 exhibited the highest volatile compound content, indicating a more intense aroma than other samples. At the same time, S20 and S25 demonstrated lower volatile compound contents, indicating a more prosperous production of volatile compounds with prolonged simmering time. Significant increases in volatile compounds were observed between S25 and S30 and between S35 and S40 (*p* < 0.05), possibly attributed to the robust Maillard reaction occurring at 25–30 min, and intensified again at 35–40 min, thereby generating a plethora of new volatile compounds. Moreover, the number of identical types of volatile compounds varied across samples, implying distinct aroma profiles (refer to Figure 5). Most volatile compounds are on the rise overall, and this phenomenon may be elucidated by the emission of inherent volatile flavor compounds from chicken and *Armillaria mellea* at the onset of pressure cooking, followed by the commencement of the Maillard reaction as pressure cooking progressed, leading to the generation of new volatile compounds. Notably, aldehydes and alcohols emerged as the most abundant compounds, pivotal in flavor changes. Previous studies have also highlighted aldehydes’ prevalence as the most abundant compounds in cooked meat [29].

The abundance of aldehydes in S40 indicates that prolonged pressure-cooking time increases aldehyde levels through lipid degradation. Concurrently, fatty alcohols resulting from lipids’ oxidative decomposition also influence flavor considerably. Furthermore, the presence of several esters, heterocyclic compounds, acids derived from aldehydes’ oxidation, and ketones from alcohol oxidation was observed, further contributing to flavor complexity.

Consistent with E-nose results, the volatile compound content of chicken with *Armillaria mellea* prepared via pressure cooking was observed with increasing pressure-cooking time (*p* < 0.05), particularly at 35–40 min. This underscores the continuous Maillard reaction between chicken with *Armillaria mellea* during the pressure-cooking process, resulting in protein degradation, lipid oxidative degradation, and augmentation in the number and content of volatile compounds, thereby imparting a distinctive and delectable aroma.

### 3.4. Characterizing the Aroma of Chicken with Armillaria mellea During the Pressure-Cooking Process Based on OAV

In order to characterize the characteristic aroma of chicken with *Armillaria mellea* prepared via pressure cooking, the OAV of each volatile flavor compound was examined using the specified odor detection threshold (Supplementary Table S2). The table lists aldehydes, alcohols, esters, acids, hydrocarbons, and ketones, totaling 45 compounds with an OAV value of at least 0.1; the compound with an OAV value of at least 1 is the sample’s primary taste ingredient. Higher OAV scores indicate a more substantial contribution of the ingredient to the overall volatile taste profile of chicken with *Armillaria mellea* prepared via pressure cooking.

#### 3.4.1. Aldehydes

Aldehydes, characteristic flavor compounds of fat breakdown and generated somewhat during amino acid degradation via the Maillard reaction, play a significant role in the overall flavor profile of chicken with *Armillaria mellea* prepared via pressure cooking due to their low odor-detection threshold and complex textures [30]. Myristic aldehyde, the primary volatile organic ingredient in chicken with *Armillaria mellea* prepared via pressure cooking, imparts a creamy, fatty flavor naturally found in chicken fat. Additionally, phenylacetaldehyde contributes a fruity and floral scent [31], benzaldehyde lends a bitter almond flavor, and hexanal adds a grassy and greasy odor [32]. These compounds are predominantly formed during the Maillard reaction of oleic, linoleic, and arachidonic acids [33], lending the aroma of chicken-cooked *Armillaria mellea* a fatty, fruity, and grassy note [34]. Nonanal, another straight-chain aldehyde present in high concentrations, exudes rose, citrus, fatty, and grassy scents and is considered a key meat flavor component [35]. Heptanal is primarily derived from lipid oxidation and Strecker breakdown of amino acids [34], imparting fermented and spicy fat odors, respectively [36]. Additionally, rapid oxidation occurs when unsaturated fatty acids are heated, generating free radicals that attack less sensitive fatty acids, such as oleic acid [34], resulting in the synthesis of octanal with a fruity, greenish scent.

#### 3.4.2. Alcohols

Alcohols can be formed via the microbial metabolism of proteins and amino acids and the reduction of ketones/aldehydes produced by lipid oxidation [36]. Straight-chain alcohols are produced by lipid oxidation [37], while most branched-chain alcohols are formed by microbial degradation of branched-chain aldehydes [38]. Despite the wide variety of alcohols produced, their relative abundance is limited, and most alcohol molecules have a high odor-detection threshold, meaning that they do not significantly impact food flavor. However, certain fatty alcohols, such as 1-octen-3-ol, trans-2-decanol, and (E)-2-nonen-1-ol, predominantly found in *Armillaria mellea*, can impart a distinctive fragrance to chicken. Among these, 1-octen-3-ol stands out due to its low odor-detection threshold and pleasant mushroom–grassy scent [39], making it a primary contributor to the taste of the samples.

#### 3.4.3. Esters

Esters are formed by the esterification of alcohols and carboxylic acids, producing highly volatile chemicals with low odor-detection thresholds [34], imparting characteristic odor attributes to chicken with *Armillaria mellea* prepared via pressure cooking. Among these, various methyl and ethyl esters, such as hexyl formate, ethyl caproate, and dipropyl phthalate, contribute to the fruity and sweet flavor of chicken with *Armillaria mellea* prepared via pressure cooking. Compounds like butyl palmitate, known for its greasy taste; dimethyl phthalate, with its aromatic odor; and butyl acetate, which imparts a butylated essence, also play significant roles in flavor development [35].

#### 3.4.4. Acids

The acids in chicken with *Armillaria mellea* prepared via pressure cooking were primarily formed through the thermal oxidation of fatty acid glycerides and phosphoric esters. Despite their presence, their high odor-detection threshold did not significantly influence the overall aroma of the dish [20], consistent with findings from E-tongue analysis.

#### 3.4.5. Hydrocarbons

Lipid oxidation during cooking led to the formation of aliphatic and aromatic hydrocarbons. Additionally, pressure cooking contributed to the partial production of alkanes from meat and fat [20]. Common alkanes detected in all samples included pentane, eicosane, and 1-methylene-4-(1-methyl phenyl) cyclohexane. Furthermore, all samples contained olefins, including n-heptene and 2-nonene. Nevertheless, due to the high odor detection-threshold values [40], they fail to impart the characteristic aroma desired for chicken with *Armillaria mellea* prepared via pressure cooking.

#### 3.4.6. Ketones

Ketones, primarily formed through lipid oxidation, the Maillard reaction, and amino acid breakdown, significantly influence the sensory properties of foods [10]. Despite their low odor-detection threshold, they possess notable organoleptic impacts. However, among the five sample groups, only floral notes of geranyl acetone, 6-methyl-5-hepten-2-one, with a pungent green, buttery aroma; and 2,5-dimethyl cyclopentanone, with a roasted aroma, were detected, along with β-methylionone, 2-methyl-2-cyclopenten-1-one, and 1-methyl-2-piperidone. Only one ketone was detected in S20 (β-methylionone) and S30 (geranyl acetone), while the other samples contained 3–4 ketones.

#### 3.4.7. Others

Furans predominantly form from the oxidation of dehydrated carbohydrates and fatty acids or through the Amadori rearrangement pathway [41]. For instance, 2-pentyl furan exhibits a caramel-like, burnt, woody flavor from fatty acid oxidation [42]. Additionally, 2-pentylfuran possesses a low odor-detection threshold and significantly contributes to the aroma of the sample. Pyrazine, a low-odor ketone produced through Strecker degradation, enriches the aroma of chicken with *Armillaria mellea* prepared via pressure cooking [20]. Indoles with a high odor-detection threshold, imparting a floral aroma [40], and certain myristic aldehydes in S30 generate myristyl ethers through heating.

The OAV of significant volatile flavor compounds notably varies across the five samples (*p* < 0.05); the cumulative OAVs of 28 compounds (key volatile flavor compounds) exceed 1. Aroma characteristics undergo considerable changes due to substantial shifts in the samples’ volatile compound content during the pressure-cooking process. While the odors of the remaining three volatile compounds have not yet been described, the aromas of these essential volatile compounds can serve as distinctive scents of chicken with *Armillaria mellea* prepared via pressure cooking. In addition, while the remaining sixteen compounds possess aromatic qualities, they function primarily as fragrance enhancers and do not exhibit the distinctive odors of chicken with *Armillaria mellea* prepared via pressure cooking. Nonanal, decanal, (E,E)-2,4-nonadienal, (E,E)-2,4-decadienal, and 1-octen-3-ol are highlighted as the main volatile compounds responsible for the volatile flavor of chicken with *Armillaria mellea* prepared via pressure cooking. These compounds have low odor-detection thresholds and relatively high relative contents. Furthermore, the balance of and interaction between volatile organic molecules can be changed by appropriately adjusting the pressure-cooking period, which will impart a distinct aroma to the samples.

### 3.5. Analysis of Differences in the Content of Volatile Compounds in Chicken with Armillaria mellea During the Pressure-Cooking Process by GC-IMS

**Figure 6:**
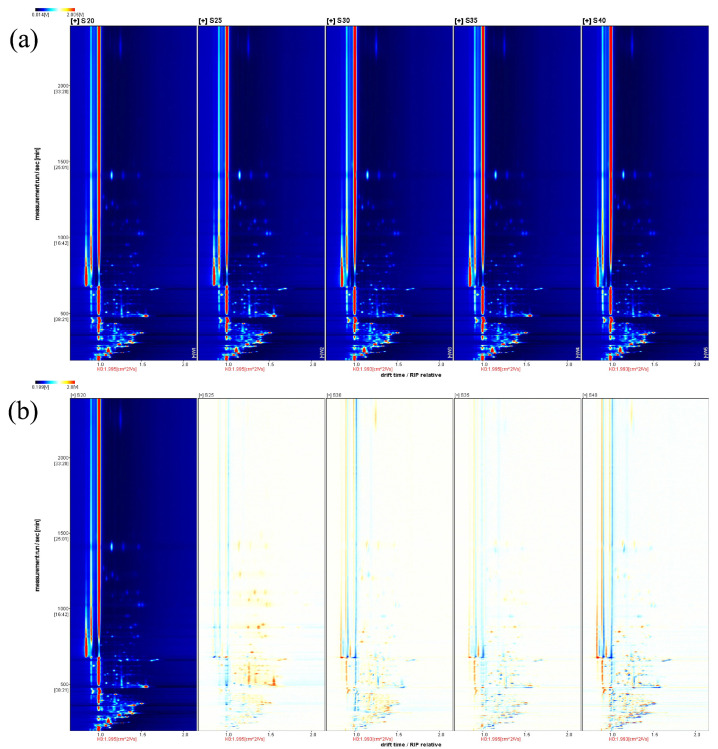
Analysis of volatile compounds, using topographic map, in chicken with *Armillaria mellea* prepared via pressure cooking. (**a**) Topographic map illustrating points on both sides of the RIP peak in the two-dimensional spectrum of volatile compounds. Color denotes the concentration of volatile compounds. (**b**) Comparison of differences in topographic maps, with compounds having concentrations similar to W 1 labeled in white and those with concentrations higher or lower than W 1 labeled in red and blue, respectively.

**Figure 7:**
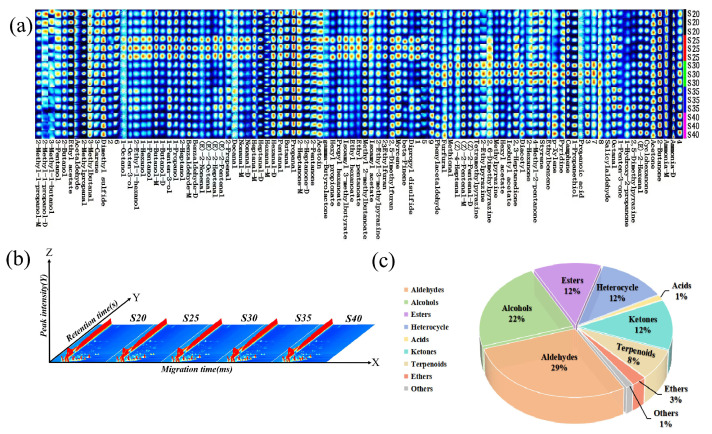
Analysis of volatile compounds, using GC-IMS, in chicken with *Armillaria mellea* prepared via pressure cooking. (**a**) Fingerprinting of volatile compounds, where each row represents the peak signal of one sample, and each column represents the same volatile compounds in other samples. Brighter colors indicate higher concentrations of volatile compounds. (**b**) The three-dimensional spectrum of volatile compounds, with the three coordinate axes representing migration time (X-axis), retention time (Y-axis), and signal peak intensity (Z-axis). (**c**) Distribution of different classes of volatile compounds.

Integrating multiple instrumental measurements is essential to obtain a more comprehensive understanding of volatile compounds. Using GC-IMS to analyze the volatile constituents of chicken with *Armillaria mellea* prepared via pressure cooking under varying pressure-cooking durations, we found thay most signals appeared within a drift time range of 0.4–1.6 (Figure 6a and Figure 7b). Although 99 peaks were detected, only 81 volatile compounds were identified, with some compounds showing multiple signals (monomers and dimers) due to ion and neutral molecule admixtures [43]. Benzaldehyde, nonanal, ammonia, heptanal, 2-heptanone, 1-butanol, (Z)-2-pentenal, 2-methyl-1-propanol, and hexanal exhibit monomers and dimers with similar retention times but different drift times (e.g., RtAmmonia monomer = 705.706 and RtAmmonia dimer = 703.863; DtAmmonia monomer = 0.91082 and DtAmmonia dimer = 0.85187). Additionally, nine signal peaks remained unidentified, comprising a total of 23 aldehydes, 18 alcohols, 10 esters, 10 heterocyclic compounds, 1 organic acid, 10 ketones, 6 terpenoids, 2 ether species, and 1 other species (Figure 7c and Supplementary Table S3). The differences in the classes and concentrations of volatile compounds detected by the two GC-based methods could be attributed to the different sampling and detection techniques. Therefore, in comparison with the GC-MS technique, only 16 volatile compounds, including phenylacetaldehyde, 1-octanol, benzaldehyde, 1-octen-3-ol, nonanal, 1-hexanol, (E)-2-heptenal, octanal, 1-pentanol, (E)-2-hexenal, heptanal, ethyl pentanoate, hexanal, pentanal, 3-methylbutanal, and decanal, were present in both methods.

To facilitate a comprehensive visual comparison of volatile compounds in chicken with *Armillaria mellea* prepared via pressure cooking across various pressure-cooking durations, we constructed fingerprints (Figure 7a) by extracting volatile compounds from the spectra [40]. Our analysis revealed higher concentrations of volatile compounds in samples S25, S30, and S40. Subsequently, S20 was used as a reference to generate a comparative plot illustrating differences among the samples (Figure 6b). Compared with S20, the remaining four groups showed either increases or decreases in compound content. Specifically, peaks in S40 and S30 displayed higher intensities and darker colors, while some compounds in S25 exhibited pronounced increases in content with darker red areas. Conversely, certain compounds in S35 demonstrated decreased content, with some areas appearing darker blue. This observation can be attributed to the continuous Maillard reaction during the pressure-cooking process. Between 25 and 30 min, the Maillard reaction intensified, generating intermediates such as ketones, aldehydes, and volatile heterocyclic compounds. Subsequently, from 35 to 40 min, the reaction underwent a renewed intensity, resulting in a complete process involving converting carbonyl and amino compounds into amino ketones, aminoketones, enamel, and pyrazine compounds. This reaction pattern aligns with trends observed in GC-MS results. Therefore, the increase in pressure-cooking time can effectively increase the number of volatile compounds in the chicken with *Armillaria mellea* prepared via pressure cooking, resulting in a more pronounced flavor profile. These findings indicated that extending the pressure-cooking duration of pressure can modulate the progression of the Maillard reaction between foods. Longer durations correspond to higher levels of volatile organic compounds. However, prolonged durations may result in the degradation of amino acids and sugars in the food, alongside the formation of carcinogenic substances [10]. Conversely, shortening the duration can suppress the Maillard reaction, extending the food’s shelf life.

### 3.6. Changes in the Texture of the Chicken with Armillaria mellea During the Pressure-Cooking Process

The texture attributes of food play a crucial role in its sensory properties and overall consumer acceptance (Table 1). Specifically, hardness, adhesiveness, and chewiness were used as key parameters to evaluate the textural properties of chicken and *Armillaria mellea*. The results demonstrated statistical significance (*p* < 0.05) in the hardness, chewiness, and adhesiveness of both chicken and *Armillaria mellea* under various pressure-cooking durations. Notably, a dramatic decrease in the hardness of both chicken and *Armillaria mellea* was observed with an extension in pressure-cooking time. This phenomenon can be attributed to variations in moisture content, protein characteristics, and loss of juices in the samples [44]. For instance, modifying myofibrillar protein structure due to oxidation facilitates the formation of new protein–protein cross-links [45].

Additionally, temperature-induced breakdown of cell-wall polysaccharides may contribute to texture softening [46]. As hardness is closely related to adhesiveness and chewiness, a significant decrease in these attributes was observed, along with a reduction in hardness (*p* < 0.05). In summary, the texture of chicken and *Armillaria mellea* can be softened by extending the simmering time. However, instrumental measurements and sensory properties are required to prevent the texture from becoming excessively loose and affecting the overall taste.

### 3.7. Sensory Properties of Chicken with Armillaria mellea During the Pressure-Cooking Process

To better understand consumers’ preferences regarding the sensory properties’ quality changes during the pressure-cooking process of chicken with *Armillaria mellea*, we selected sensory evaluation with 12 panelists by scoring samples across four aspects: appearance, odor, taste, and flavor. Table 2 presents the evaluation outcomes, with each sample demonstrating statistical significance (*p* < 0.05). The overall appearance score difference between the samples was slight because only the cooking time was changed, and the chicken-to-*Armillaria mellea* material ratio was unchanged. The pressure-cooking time does not affect the product’s appearance [47]. However, significant effects on taste and smell were noted (*p* < 0.05). The mushroom aroma, fat aroma, baked-butter aroma, and floral and fruity aroma became more assertive with the extension of pressure-cooking time. This could be attributed to the Maillard and lipid oxidation reactions between meat and *Armillaria mellea* during the pressure-cooking process, which generates volatile compounds such as aldehydes, alcohols, and hydrocarbons [48,49]. Notably, S35 received the highest flavor rating, likely due to the gradual loosening of muscle fibers in the pressured environment. Conversely, prolonged cooking times, as in S40, resulted in excessively chewy and loose fibers, resembling minced meat and receiving lower scores from panelists. In general, the sensory properties’ scores matched the instrumental results. For example, E-tongue was found to have a high level of pleasant freshness at 40 min, and taste was also the highest score at 40 min. The GC-MS, GC-IMS, and E-nose all found a significant increase in volatile compounds at 40 min, and flavor also received the highest score at 40 min. Notably, chicken with *Armillaria mellea* prepared via pressure cooking scored highest at 35–40 min, suggesting consumer preference and providing a theoretical basis for future industrial production of pre-prepared dishes featuring this delicacy.

## 4. Conclusions

This study is the first to investigate the effects of the interaction between chicken and *Armillaria mellea* on the number of volatile compounds and sensory properties during pressure cooking. This study aims to fully understand the dynamic changes in chicken with *Armillaria mellea* during the pressure-cooking process and maximize its potential for product development. E-tongue analysis found that the extension of pressure-cooking time significantly affected taste, especially umami. E-nose, GC-MS, and GC-IMS combined found that the number of volatile compounds fluctuated significantly with the length of pressure-cooking time. In the OAV analysis of volatile compounds, nonanal, decanal, (E,E)-2,4-nonadienal, (E,E)-2,4-decadienal, and 1-octen-3-ol were considered to be the most important aroma contributors, which gave the chicken and *Armillaria mellea* their unique fat, oil, and mushroom aromas. Regarding the texture and sensory properties, we found that the taste significantly improved with the extension of pressure-cooking time. However, the overall texture of chicken with *Armillaria mellea* prepared via pressure cooking was affected when the pressure-cooking time reached 40 min. In summary, these findings reveal the flavor changes in chicken with *Armillaria mellea* during the pressure-cooking process. Determining the ideal pressure-cooking time of 35 to 40 min provides a theoretical basis for preparing high-quality, ready-to-eat chicken with Armillaria mellea prepared via pressure cooking. This study is the first to explore complex substances and the dynamic changes in the mutual flavor substances between the two substances at different pressure-cooking stages. This study also encourages people to explore the effects of heat treatment on food flavor and taste and the development of complex food flavors. However, it is important to note that the traceability of these substances has certain limitations. In future studies, it will be crucial to delve into the underlying processes of taste precursor alterations during pressure cooking and explore how various cooking methods impact the production of volatile compounds in chicken with Armillaria mellea prepared via pressure cooking.

## Figures and Tables

**Table 1:** Textural characteristics of chicken and *Armillaria mellea* treated with different pressure-cooking times.

	Chicken			*Armillaria mellea*		
	Hardness (N)	Chewiness (mJ)	Gumminess (N)	Hardness (N)	Chewiness (mJ)	Gumminess (N)
S20	10,454.85 ± 88.73 ^a^	2515.31 ± 34.94 ^a^	3978.95 ± 6.34 ^a^	576.93 ± 27.10 ^a^	402.27 ± 13.61 ^a^	431.79 ± 8.94 ^a^
S25	9534.33 ± 327.22 ^b^	2223.62 ± 39.28 ^b^	3735.86 ± 31.35 ^b^	397.82 ± 7.66 ^b^	305.25 ± 13.34 ^b^	361.20 ± 24.11 ^b^
S30	8944.59 ± 441.60 ^b^	1977.69 ± 31.74 ^c^	3417.52 ± 57.65 ^c^	355.91 ± 17.84 ^c^	249.02 ± 1.13 ^c^	276.61 ± 4.47 ^c^
S35	8110.51 ± 480.06 ^c^	1550.80 ± 89.17 ^d^	2863.10 ± 94.77 ^d^	307.51 ± 36.47 ^d^	185.43 ± 4.92 ^d^	216.52 ± 3.08 ^d^
S40	7238.34 ± 222.03 ^d^	1244.36 ± 76.26 ^e^	2662.67 ± 94.57 ^e^	256.34 ± 6.36 ^e^	157.28 ± 2.58 ^e^	194.08 ± 0.06 ^d^

The data are presented as the mean ± SD. Different letters within the same column indicate significant differences (*p* < 0.05).

**Table 2:** Sensory properties of chicken and *Armillaria mellea* treated with different pressure-cooking times.

	Appearance	Taste	Texture	Flavor	Total Score
S20	18.05 ± 1.67 ^d^	18.20 ± 1.47 ^e^	17.35 ± 1.76 ^d^	19.00 ± 1.59 ^e^	72.60 ± 3.28 ^e^
S25	19.25 ± 1.21 ^c^	19.90 ± 0.91 ^d^	19.05 ± 1.23 ^c^	20.25 ± 1.29 ^d^	78.45 ± 3.09 ^d^
S30	19.70 ± 1.03 ^bc^	21.00 ± 0.92 ^c^	20.40 ± 1.47 ^b^	21.20 ± 1.64 ^c^	82.30 ± 2.89 ^c^
S35	20.20 ± 1.24 ^ab^	22.25 ± 1.55 ^b^	23.05 ± 1.28 ^a^	22.30 ± 1.38 ^b^	87.80 ± 3.79 ^a^
S40	20.95 ± 1.85 ^a^	23.90 ± 1.07 ^a^	19.40 ± 1.76 ^c^	23.70 ± 1.22 ^a^	87.75 ± 2.96 ^a^

The data are presented as the mean ± SD. Different letters within the same column indicate significant differences (*p* < 0.05).

## Data Availability

The data used to support the findings of this study can be made available by the corresponding author upon request.

## Supplementary Materials

The following supporting information can be downloaded at https://www.mdpi.com/article/10.3390/foods14010083/s1, Table S1: Identification of volatile chemicals in the chicken with *Armillaria mellea* during the pressure-cooking process based on GC-MS.; Table S2: OAV of volatile compounds in chicken with *Armillaria mellea* prepared via pressure cooking for different pressure-cooking times; Table S3: Identification of volatile chemicals in the chicken with *Armillaria mellea* during the pressure-cooking process based on GC-IMS.
