# Supplementary material for: Changes in Volatile Compounds and Sensory Properties of Chicken with Armillaria mellea During the Pressure-Cooking Process
Source: Foods. 2025 Jan 1;14(1):83. doi: 10.3390/foods14010083 (PMC11719872; doi:10.3390/foods14010083)
Supplement: Supplementary file 1 [file foods-14-00083-s001.zip › Supplementary Tables.pdf]

Table S1. Identification of volatile chemicals in the chicken with *Armillaria mellea* during the pressure cooking process based on GC-MS.

	CAS	chemical formula	RI	Compound	S20	S25	S30	S35	S40
				Aldehyde (28)					
6.41	7779-41-1	$C_{12}H_{26}O_2$	1366	1,1-dimethoxydecane	nd	nd	nd	53.23±3.38^{b}	85.00±16.68^{a}
6.48	101-48-4	$C_{10}H_{14}O_2$	1222	(2,2-Dimethoxyethyl) benzene	nd	68.46±3.17^{b}	nd	52.39±4.00^{c}	90.49±0.40^{a}
7.04	10022-28-3	$C_{10}H_{22}O_2$	1178	Octanal dimethyl acetal	nd	nd	nd	nd	69.26±4.79
7.06	110-62-3	$C_5H_{10}O$	699	pentanal	34.55±2.34	nd	nd	nd	nd
7.10	107-75-5	$C_{10}H_{20}O_2$	1300	3,7-Dimethyl-7-hydroxyoctanal	27.51±3.40^{a}	19.47±0.95^{c}	24.29±0.09^{b}	nd	nd
7.33	66-25-1	$C_6H_{12}O$	800	Hexanal	402.48±70.38^{b}	114.09±0.85^{d}	140.63±4.92^{cd}	186.59±19.98^{c}	1352.05±30.75^{a}
7.46	124-13-0	$C_8H_{16}O$	1003	octanal	237.7±13.20^{c}	309.44±35.95^{b}	168.00±23.15^{d}	149.48±5.30^{d}	492.53±31.38^{a}
7.62	112-45-8	$C_{11}H_{20}O$	1288	10-Undecenal	31.56±0.69^{c}	39.80±0.70^{b}	nd	48.89±4.80^{a}	nd
7.68	124-19-6	$C_9H_{18}O$	1104	1-Nonanal	583.81±41.72^{b}	374.36±6.96^{c}	247.76±26.12^{d}	192.31±5.13^{e}	2057.70±35.88^{a}
7.78	112-31-2	$C_{10}H_{20}O$	1206	Decanal	nd	nd	175.01±28.31	168.05±8.76	166.69±27.69
8.15	1620-98-0	$C_{15}H_{22}O_2$	1772	3,5-Di-tert-butyl-4-hydroxybenzaldehyde	nd	nd	nd	nd	27.65±8.34
8.75	6728-26-3	$C_6H_{10}O$	854	trans-2-Hexenal	50.57±0.80^{a}	16.99±0.89^{c}	29.21±6.31^{b}	51.60±9.11^{a}	19.00±0.60^{c}
9.40	14371-10-9	C_9H_8O	1270	trans-Cinnamaldehyde	nd	nd	24.37±3.47	nd	nd
10.08	104-67-6	$C_{11}H_{20}O_2$	1576	Peach aldehyde	54.04±3.05^{b}	68.49±5.55^{a}	32.61±5.83^{d}	40.60±4.62^{c}	43.61±8.09^{c}
10.12	104-61-0	$C_9H_{16}O_2$	1363	gamma-Nonanolactone	89.68±5.71^{b}	nd	105.55±2.81^{a}	nd	nd
10.24	112-54-9	$C_{12}H_{24}O$	1409	Lauraldehyde	142.37±19.9^{a}	108.35±3.91^{b}	155.65±12.09^{a}	102.62±7.39^{b}	93.03±0.90^{b}
10.38	5392-40-5	$C_{10}H_{16}O$	1276	Citral	nd	nd	18.98±1.19	nd	nd
12.42	5910-87-2	$C_9H_{14}O$	1216	trans,trans-2,4-Nonadienal	63.91±8.36^{bc}	49.96±7.18^{bc}	42.71±8.10^{c}	70.71±13.82^{b}	153.43±21.92^{a}

13.90	590-86-3	$C_5H_{10}O$	652	Isovaleraldehyde	nd	nd	101.47±0.94[c]	142.17±11.12[a]	127.56±9.81[b]
20.13	111-71-7	$C_7H_{14}O$	901	Heptaldehyde	55.71±1.67[d]	62.85±2.56[d]	149.29±1.27[b]	71.56±4.62[c]	178.64±7.66[a]
20.73	100-52-7	C_7H_6O	962	Benzaldehyde	650.85±29.31[c]	507.98±30.47[d]	860.24±8.63[b]	855.46±64.14[b]	2181.71±77.51[a]
21.91	101-49-5	$C_{10}H_{12}O_2$	1277	2-Benzyl-1,3-dioxolane	nd	59.85±6.60[c]	73.58±5.53[a]	64.59±4.68[bc]	70.76±6.98[ab]
22.73	122-78-1	C_8H_8O	1045	Phenylacetaldehyde	177.19±5.85[c]	155.65±26.07[c]	299.01±9.09[b]	202.81±8.44[c]	469.37±56.46[a]
24.85	25152-84-5	$C_{10}H_{16}O$	1317	(E,E)-2,4-Decadien-1-al	101.95±14.35[b]	68.09±6.01[c]	105.16±1.49[b]	60.98±1.51[c]	136.00±0.91[a]
26.31	21834-92-4	$C_{13}H_{16}O$	1486	5-methyl-2-phenylhex-2-enal	nd	nd	nd	nd	67.82±10.50
27.85	112-44-7	$C_{11}H_{22}O$	1307	Undecanal	74.56±8.19[b]	58.95±5.04[c]	74.55±6.75[b]	70.37±8.42[bc]	118.17±7.23[a]
28.22	124-25-4	$C_{14}H_{28}O$	1613	Tetradecanal	669.60±30.8[c]	669.87±18.95[c]	824.32±54.5[b]	1794.75±94.73[a]	1803.25±61.69[a]
30.17	629-80-1	$C_{16}H_{32}O$	1817	Hexadecanal	459.24±40.47[c]	183.01±5.81[d]	436.96±5.02[c]	803.56±55.62[a]	520.65±19.00[b]
					Alcohol (23)				
6.21	623-39-2	$C_4H_{10}O_3$	977	3-Methoxy-1,2-propanediol	57.00±6.45a	20.19±1.34d	41.66±8.98[b]	31.73±0.28[c]	39.23±1.66[bc]
6.52	141-92-4	$C_{12}H_{26}O_3$	1414	8,8-Dimethoxy-2,6-dimethyloctan-2-ol	nd	nd	nd	52.82±1.49[b]	63.06±3.48[a]
7.28	928-91-6	$C_6H_{12}O$	879	(Z)-Hex-4-en-1-ol	nd	nd	nd	nd	35.40±9.23
7.64	31502-14-4	$C_9H_{18}O$	1176	(E)-Non-2-en-1-ol	29.88±0.35[c]	89.96±0.44[a]	31.59±3.69[c]	nd	49.54±4.06[b]
7.67	928-95-0	$C_6H_{12}O$	862	(E)-hex-2-enol	nd	nd	nd	74.58±9.85[b]	1127.74±82.82[a]
7.76	1576-95-0	$C_5H_{10}O$	767	cis-2-penten-1-ol	nd	nd	nd	64.25±8.21[b]	70.60±2.09[a]
7.96	111-27-3	$C_6H_{14}O$	868	Hexyl alcohol	293.34±21.64[a]	202.44±0.09[b]	138.07±11.59[c]	113.34±5.03[d]	91.03±8.93[e]
8.59	71-41-0	$C_5H_{12}O$	765	1-Pentanol	70.12±8.51[c]	167.20±29.25[a]	112.80±7.30[b]	112.25±5.34[b]	nd
11.07	2051-31-2	$C_{10}H_{22}O$	1182	4-decanol	nd	nd	nd	45.97±0.35[a]	41.91±4.01[b]
12.76	111-87-5	$C_8H_{18}O$	1071	1-Octanol	58.08±6.85[a]	32.12±5.30[bc]	21.51±2.92[c]	37.87±3.68[b]	68.17±8.74[a]
13.3	3690-05-9	$C_9H_{10}O_2$	1639	4-hydroxycinnamyl alcohol	nd	42.12±4.94[b]	nd	30.64±4.60[c]	61.48±7.48[a]

13.99	372-66-7	$C_8H_{19}N0$	1099	6-amino-2-methylheptan-2-ol	81.17±8.90[a]	nd	25.55±1.27[c]	nd	37.92±4.46[b]
14.25	6982-25-8	$C_4H_{10}O_2$	773	2,3-butanediol	nd	nd	51.09±4.88[b]	64.96±7.30[b]	183.47±18.89[a]
15.08	18409-18-2	$C_{10}H_{20}O$	1257	(E)-2-Decenol	57.68±12.31[a]	25.17±0.76[b]	69.90±9.74[a]	36.99±9.67[b]	61.30±4.92[a]
15.57	628-99-9	$C_9H_{20}O$	1102	2-Nonanol	nd	nd	44.43±9.87[b]	48.83±2.74[b]	74.55±3.41[a]
16.49	764-01-2	C_4H_6O	695	2-Butyn-1-ol	nd	nd	43.58±2.59	nd	nd
17.72	143-08-8	$C_9H_{20}O$	1173	1-nonanol	145.85±8.34[d]	157.26±35.14[cd]	201.10±8.60[bc]	231.01±4.72[b]	369.88±45.90[a]
18.64	106-22-9	$C_{10}H_{20}O$	1228	Citronellol	119.94±21.39[c]	96.42±8.70[d]	129.55±6.71[c]	181.27±7.17[b]	433.40±6.74[a]
18.84	502-41-0	$C_7H_{14}O$	1022	Cycloheptanol	nd	nd	nd	53.61±1.54	50.80±4.59
19.29	3391-86-4	$C_8H_{16}O$	980	Mushroom alcohol	148.09±2.97[b]	55.42±1.69[d]	126.43±9.18[b]	91.41±8.12[c]	255.45±28.81[a]
19.90	78-69-3	$C_{10}H_{22}O$	1100	Tetrahydrolinalool	64.63±6.00	46.78±0.87	72.77±2.15[bc]	87.58±1.50[b]	185.03±22.13[a]
23.00	504-01-8	$C_6H_{12}O_2$	1092	1,3-Cyclohexanediol	nd	nd	nd	nd	376.41±3.95
26.04	1679-51-2	$C_7H_{12}0$	1106	3-Cyclohexene-1-methanol	158.34±4.06[b]	73.98±0.18[c]	nd	60.41±4.96[c]	217.97±12.78[a]
					Ester (13)				
6.56	106-36-5	$C_6H_{12}O_2$	807	Propyl propionate	nd	71.04±7.71	nd	nd	nd
6.89	5185-97-7	$C_7H_{12}O_3$	1053	4-Oxopentyl acetate	nd	nd	nd	nd	42.50±9.07
7.83	589-66-2	$C_8H_{14}O_2$	983	2-Butenoic acid, 2-methylpropyl ester	91.33±9.3[b]	199.61±20.36[a]	nd	nd	nd
8.12	1499-53-2	$C_{11}H_{13}NO_3$	1726	Glycine, N-benzoyl-,ethyl ester	57.21±8.09[a]	33.67±9.59[b]	50.27±4.47[a]	29.42±0.29[b]	55.22±7.57[a]
11.70	131-16-8	$C_{14}H_{18}O_4$	1756	Dipropyl phthalate	222.23±4.76[b]	229.70±7.02[b]	74.37±0.07[d]	132.90±3.95[c]	287.37±0.03[a]
11.73	131-18-0	$C_{18}H_{26}O_4$	2129	1,2-Benzenedicarboxylic acid, dipentyl ester	nd	49.63±9.24[a]	nd	22.36±0.25[b]	46.08±4.80[a]
12.86	93-28-7	$C_{12}H_{14}O_3$	1524	Phenol,2-methoxy-4-(2-propen-1-yl)-, 1-acetate	80.33±5.15[a]	36.01±6.53[c]	21.82±0.74[d]	38.82±0.09[c]	58.20±1.21[b]
13.17	105-86-2	$C_{11}H_{18}O_2$	1300	Geranyl formate	66.93±3.58[bc]	82.97±7.01[b]	57.39±8.12[c]	83.38±15.83[b]	190.08±6.11[a]

16.24	5396-64-5	$C_{12}H_{14}O_4$	1873	3,4-O-Dimethylcaffeic acid methyl ester	118.69±16.34^{cd}	84.82±6.39^{d}	151.66±23.38^{bc}	159.37±6.43^{b}	307.69±33.05^{a}
18.27	2566-89-4	$C_{21}H_{34}O_2$	2274	Methylall-cis-5,8,11,14-eicosa tetraenoate	38.34±1.53^{b}	16.40±0.04^{b}	27.50±4.67^{b}	43.55±9.00^{b}	151.56±31.97^{a}
22.23	629-33-4	$C_7H_{14}O_2$	914	Hexyl formate	nd	nd	57.71±1.08	nd	nd
29.91	131-11-3	$C_{10}H_{10}O_4$	1454	Dimethyl phthalate	428.00±35.20^{d}	383.29±3.97^{d}	900.06±43.00^{b}	641.40±20.45^{c}	1403.35±0.23^{a}
35.23	111-06-8	$C_{20}H_{40}O_2$	2188	butyl palmitate	336.35±30.64^{a}	107.49±17.87^{d}	181.23±6.03^{b}	143.45±9.79^{c}	204.14±2.47^{b}
				Acid (9)					
9.58	373-49-9	$C_{16}H_{30}O_2$	1951	Oleopalmitic acid	nd	nd	30.46±0.31^{b}	nd	43.28±8.46^{a}
14.83	59-30-3	$C_{19}H_{19}N_7O_6$	1387	Folic acid	227.39±26.85^{b}	172.38±23.61^{c}	117.91±1.78^{d}	220.38±14.33^{b}	275.31±4.68^{a}
19.52	127-17-3	$C_3H_4O_3$	1249	Pyruvic acid	148.03±24.58^{ab}	107.56±5.79^{c}	152.25±11.09^{ab}	123.92±18.46^{bc}	163.06±16.27^{a}
19.66	64-19-7	$C_2H_4O_2$	610	Acetic acid glacial	167.65±8.62^{b}	138.80±23.77^{c}	205.46±1.80^{a}	115.96±0.63^{d}	113.11±7.07^{d}
24.18	2305-36-4	$C_8H_9NO_2$	949	2-Amino-4-methylbenzoic acid	391.43±34.11^{b}	242.97±3.71^{c}	205.48±1.12^{cd}	195.08±30.95^{d}	447.75±22.55^{a}
25.29	142-62-1	$C_6H_{12}O_2$	990	Hexanoic acid	175.00±4.13^{b}	90.20±2.84^{c}	176.04±27.18^{b}	181.01±29.50^{b}	484.27±59.83^{a}
28.66	57-10-3	$C_{16}H_{32}O_2$	1968	Palmitic acid	583.89±46.55^{b}	226.06±50.94^{d}	473.41±32.01^{c}	934.21±42.41^{a}	913.35±30.81^{a}
30.4	112-80-1	$C_{18}H_{34}O_2$	2141	Oleic acid	134.74±7.01^{d}	177.59±10.84^{c}	322.37±1.44^{a}	196.03±1.03^{b}	310.19±7.61^{a}
33.68	506-30-9	$C_{20}H_{40}O_2$	2365	Eicosanoic acid	173.00±1.88^{a}	137.75±23.26^{b}	155.05±6.05^{ab}	164.25±1.28^{a}	164.71±13.25^{a}
				Heterocyclic compound (11)					
8.89	6090-09-1	$C_9H_{14}O$	1137	4-acetyl-1-methyl-1-cyclohex ene	nd	nd	30.67±5.47	nd	nd
10.18	2305-05-7	$C_{12}H_{22}O_2$	1678	gamma-Dodecalactone	56.62±1.91^{b}	59.62±3.84^{b}	62.39±8.89^{b}	56.53±0.41^{b}	94.45±7.38^{a}
11.59	1139-30-6	$C_{15}H_{24}O$	1581	caryophyllene oxide	nd	50.98±1.95^{b}	nd	109.80±13.31^{a}	61.06±6.82^{b}
12.14	4466-24-4	$C_8H_{12}O$	893	2-Butylfuran	126.88±4.67^{b}	73.41±1.95^{c}	70.05±5.34^{c}	124.81±0.10^{b}	161.63±30.42^{a}

12.24	3777-69-3	$C_9H_{14}O$	993	2-Pentylfuran	66.18±2.69[bc]	74.69±21.39[b]	73.53±5.62[b]	49.90±1.14[c]	153.80±8.43[a]
20.56	613-13-8	$C_{14}H_{11}N$	2234	2-Anthracenamine	nd	nd	291.93±14.32[a]	246.24±13.45[b]	nd
22.52	142-68-7	$C_5H_{10}O$	714	Tetrahydropyran	193.89±7.26[b]	183.04±7.47[b]	226.29±3.14[ab]	211.81±52.89[ab]	260.28±21.75[a]
22.95	24295-03-2	C_5H_5NOS	1022	2-Acetylthiazole	122.95±8.75[b]	62.68±7.57[c]	153.81±6.74[a]	59.45±0.18[c]	32.47±4.56[d]
23.48	16400-72-9	$C_{12}H_{20}O_2$	1704	2H-Pyran-2-one,6-heptyl-5,6-dihydro	72.91±10.61[c]	56.43±7.37[d]	98.19±1.18[b]	65.29±6.69[cd]	163.13±9.19[a]
27.42	1927-68-0	$C_9H_{18}O_2$	1100	2-Butoxytetrahydro-2H-pyran	64.63±6.00[c]	124.43±2.09[b]	280.87±6.94[a]	125.92±17.40[b]	261.27±18.78[a]
31.39	580-49-4	$C_{17}H_{22}O_5$	2383	Xanthumin	481.33±2.72[c]	669.71±57.54[a]	575.86±38.76[b]	564.55±42.73[b]	639.19±51.89[ab]
				Ketone (6)					
6.32	931-20-4	$C_6H_{11}NO$	1178	1-Methyl-2-Piperidone	nd	75.37±8.91[b]	nd	nd	84.49±6.49[a]
9.69	3796-70-1	$C_{13}H_{22}O$	1453	Geranyl acetone	nd	nd	46.63±7.23	nd	nd
9.92	127-43-5	$C_{14}H_{22}O$	1584	β-Methylionone	33.89±5.97[b]	23.98±1.06[b]	nd	31.37±3.71[b]	58.19±9.68[a]
13.44	1120-73-6	C_6H_8O	913	2-methyl-2-cyclopenten-1-one	nd	52.34±8.84[b]	nd	60.17±8.47[b]	178.46±3.77[a]
15.19	110-93-0	$C_8H_{14}O$	986	6-Methyl-5-hepten-2-one	nd	nd	nd	49.16±1.41[b]	76.71±8.67[a]
22.64	4041-09-2	$C_7H_{12}O$	855	2,5-Dimethyl cyclopentan-1-one	nd	nd	nd	69.62±7.85	nd
				Hydrocarbon (5)					
6.68	109-66-0	C_5H_{12}	500	Pentane	175.88±15.07[a]	77.54±15.71[d]	51.67±9.38[e]	107.31±3.74[c]	130.52±8.70[b]
16.54	592-76-7	C_7H_{14}	685	1-Heptene	48.75±1.19[d]	62.32±3.05[c]	34.18±3.37[e]	76.87±0.68[b]	85.59±0.96[a]
19.38	112-95-8	$C_{20}H_{42}$	2000	Eicosane	54.22±2.58[b]	24.63±0.43[c]	49.72±9.33[b]	51.16±3.00[b]	177.26±5.67[a]
21.32	6434-78-2	C_9H_{18}	897	(E)-2-Nonene	nd	22.20±4.56[d]	140.04±7.11[b]	80.58±8.89[c]	202.53±18.52[a]
26.03	499-97-8	$C_{10}H_{16}$	1004	1-Isopropenyl-4-methylenecyclohexane	nd	nd	137.43±6.06	nd	nd
				Amine (4)					

6.63	105-41-9	$C_7H_{17}N$	1152	1,3-Dimethylpentylamine	56.65±3.92^b	33.77±3.14^c	24.33±0.55^d	51.29±5.34^b	80.85±7.93^a
8.84	926-63-6	$C_5H_{13}N$	597	N,N-Dimethylpropylamine	43.04±1.92^b	21.54±0.38^d	28.64±0.28^c	52.54±4.43^a	42.50±3.31^b
11.56	2439-54-5	$C_9H_{21}N$	1088	N-Methyloctylamine	79.66±5.8^a	nd	nd	nd	43.82±1.65^b
26.75	35205-54-0	$C_9H_{13}NO$	1226	1-phenoxy-2-propanamin	nd	nd	60.48±9.29	nd	nd
				Ether (1)					
8.97	607-91-0	$C_{11}H_{12}O_3$	1519	Myristicin	nd	nd	23.83±1.65	nd	nd
				Other (1)					
31.28	120-72-9	C_8H_7N	1295	Indole	139.60±4.25^a	50.36±5.99^b	147.8±9.17^a	139.16±17.64^a	155.31±22.90^a

nd, not determined; The data are presented as the mean±SD; Different letters within the same row indicate significant differences ($p < 0.05$).

Table S2. OAV of volatile compounds in chicken with *Armillaria mellea* by pressure cooking treated with different pressure cooking times

Volatile compounds		odor threshold value (μg/kg)	Odor description	S20	S25	S30	S35	S40
Aldehyde	(2,2-Dimethoxyethyl) benzene	0.6	nd	nd	114.11±5.28[b]	nd	87.32±6.67[c]	150.82±0.67[a]
	3,7-Dimethyl-7-hydroxyoctanal	0.01	Green, Grass	3987.23±492.05[a]	2821.47±137.18[c]	3520.31±12.88[b]	nd	nd
	Hexanal	4.5	Apple, fat, fresh, green, oil	89.44±15.64[b]	25.35±0.19[d]	33.47±1.09[cd]	41.47±4.44[c]	300.46±6.83[a]
	octanal	0.7	Citrus, fat, green, oil, pungent	339.57±18.85[c]	442.06±51.36[b]	240.00±33.07[d]	213.54±7.57[d]	703.62±44.83[a]
	1-Nonanal	1	Floral, fatty, green, lemon-like	583.81±41.72[b]	374.36±6.96[c]	247.76±26.12[d]	192.31±5.13[e]	2057.70±35.88[a]
	Decanal	0.1	Faint scent	nd	nd	1750.13±283.12	1680.49±87.57	1666.88±276.93
	trans-2-Hexenal	82	Cheese	0.62±0.01[a]	0.21±0.01[c]	0.36±0.08[b]	0.63±0.11[a]	0.23±0.01[c]
	trans-Cinnamaldehyde	600	Cinnamon	nd	nd	0.04±0.01	nd	nd
	Peach aldehyde	2.1	Peach	25.73±1.45[b]	32.61±2.64[a]	nd	19.33±2.20[c]	20.77±3.85[c]
	gamma-Nonanolactone	9.7	Coconut	9.25±0.59[b]	nd	10.88±0.29[a]	nd	nd
	Lauraldehyde	2	Oil	71.18±9.95[a]	54.18±1.95[b]	77.83±6.04[a]	51.31±3.69[b]	46.52±0.45[b]
	Citral	28	Lemon, sweetness	nd	nd	0.68±0.04	nd	nd
	(E,E)-2,4-Nonadienal	0.06	Fat	1065.14±139.36[bc]	832.68±119.64[bc]	711.92±135.06[c]	1178.43±230.27[b]	2557.19±365.30[a]

	Isovaleraldehyde	0.2	Malty, fruity	nd	nd	507.37±4.69^{c}	710.83±55.58^{a}	637.80±49.07^{b}
	Heptaldehyde	3	Soap, herbal	18.57±0.56^{d}	20.95±0.85^{d}	49.76±0.42^{b}	23.85±1.54^{c}	59.55±2.55^{a}
	Benzaldehyde	350	Bitter almond, cherry	1.86±0.08^{c}	1.45±0.09^{d}	2.46±0.02^{b}	2.44±0.18^{b}	6.23±0.22^{a}
	Phenylacetaldehyde	4	Fruity	44.30±1.46^{c}	38.91±6.52^{c}	74.75±2.27^{b}	50.70±2.11^{c}	117.34±14.12^{a}
	(E,E)-2,4-Decadien-1-al	0.13	Oil	784.20±110.39^{b}	523.76±46.24^{c}	808.96±11.43^{b}	469.10±11.65^{c}	1046.17±6.97^{a}
	Undecanal	12.5	nd	5.96±0.66b	4.72±0.40^{c}	5.96±0.54^{b}	5.63±0.67^{bc}	9.45±0.58^{a}
	Tetradecanal	160	Creamy, fat	4.19±0.19^{c}	4.19±0.12^{c}	5.15±0.34^{b}	11.22±0.59^{a}	11.27±0.39^{a}
Alcohol	(E)-Non-2-en-1-ol	130	Citrus-like, vegetable	0.23±0.00^{c}	0.69±0.00^{a}	0.24±0.03^{c}	nd	0.38±0.03^{b}
	Hexyl alcohol	250	Fruit, banana-like, soft	1.17±0.09^{a}	0.81±0.00^{b}	0.55±0.05^{c}	0.45±0.02^{d}	0.36±0.04^{e}
	1-Pentanol	2540	Fruity	0.03±0.00^{c}	0.07±0.01^{a}	0.04±0.00^{b}	0.04±0.00^{b}	nd
	1-Octanol	110	Green, citrus-like, fatty, coconut	0.53±0.06^{a}	0.29±0.05^{bc}	0.20±0.03^{c}	0.34±0.03^{b}	0.62±0.08^{a}
	2-Nonanol	58	Cucumber	nd	nd	0.77±0.17^{b}	0.84±0.05^{b}	1.29±0.06^{a}
	1-nonanol	2	Fat, Floral, Green, Oil	72.92±4.17^{d}	78.63±17.57^{cd}	100.55±4.30^{bc}	115.50±2.36^{b}	184.94±22.95^{a}
	Citronellol	10.6	Rose	11.31±2.02^{c}	9.10±0.82^{d}	12.22±0.63^{c}	17.10±0.68^{b}	40.89±0.64^{a}
	Cycloheptanol	4800	nd	nd	nd	nd	0.01±0.00	0.01±0.00
	Mushroom alcohol	1	Mushroom	148.09±2.97^{b}	55.42±1.69^{d}	126.43±9.18^{b}	91.41±8.12^{c}	255.45±28.81^{a}
Ester	Geranyl formate	200	Floral	0.33±0.02^{cd}	0.41±0.04^{bc}	0.29±0.04^{d}	0.42±0.08^{b}	0.95±0.03^{a}
Acid	Acetic acid glacial	22000	Sour	0.01±0.00	0.01±0.00	0.01±0.00	0.01±0.00	0.01±0.00

	Hexanoic acid	92	Oil, milk	1.90±0.04^{b}	0.98±0.03^{c}	1.91±0.30^{b}	1.97±0.32^{b}	5.26±0.65^{a}
	Oleic acid	44000	Lard odor	nd	nd	0.01±0.00	nd	0.01±0.00
	Eicosanoic acid	20000	Fat	0.01±0.00	0.01±0.00	0.01±0.00	0.01±0.00	0.01±0.00
	gamma-Dodecalactone	7	Creamy, fruity	8.09±0.27^{b}	8.52±0.55^{b}	8.91±1.27^{b}	8.08±0.06^{b}	13.49±1.05^{a}
Heterocyclic compounds	2-Butylfuran	5	nd	25.38±0.93^{b}	14.68±0.39^{c}	14.01±1.07^{c}	24.96±0.02^{b}	32.33±6.08^{a}
	2-Pentylfuran	6	Caramel-like, cooked, woody	11.03±0.45^{bc}	12.45±3.57^{b}	12.25±0.94^{b}	8.32±0.19^{c}	25.63±1.41^{a}
	2-Acetylthiazole	4	nd	30.74±2.19^{b}	15.67±1.89^{c}	38.45±1.68^{a}	14.86±0.05^{c}	8.12±1.14^{d}
Ketone	Geranyl acetone	60	Floral	nd	nd	0.78±0.12	nd	nd
	6-Methyl-5-hepten-2-one	50	Pungent, green	nd	nd	nd	0.98±0.03^{b}	1.53±0.17^{a}
	2,5-Dimethylcyclopentan-1-one	90	Buttery, toasty	nd	nd	nd	0.77±0.09	nd
Hydrocarbon	Pentane	4100	nd	0.04±0.00^{a}	0.02±0.00^{c}	0.01±0.00^{d}	0.03±0.00^{b}	0.03±0.00^{b}
	1-Heptene	1500	nd	0.03±0.00^{d}	0.04±0.00^{c}	0.02±0.00^{e}	0.05±0.00^{b}	0.06±0.00^{a}
Ether	Myristicin	25	Fat, oil	nd	nd	0.95±0.07	nd	nd
Other	Indole	140	Floral	1.00±0.03^{a}	0.36±0.04^{b}	1.06±0.07^{a}	0.99±0.13^{a}	1.11±0.16^{a}

nd, not determined; The data are presented as the mean±SD; Different letters within the same row indicate significant differences ($p < 0.05$).

Table S3. Identification of volatile chemicals in the chicken with *Armillaria mellea* during the pressure cooking process based on GC-IMS.

	RI	Rt [sec]	Dt [RIPrel]	S20	S25	S30	S35	S40
Salicylaldehyde	1776.10	2274.47	1.14	1128.54±122.09	1181.12±59.88	1226.29±25.80	1206.91±30.96	1112.58±14.16
Phenylacetaldehyde	1769.60	2242.67	1.26	3658.40±97.72[c]	3711.45±156.33[c]	4803.92±181.79[a]	3385.82±273.31[c]	4369.21±252.69[b]
gamma-Butyrolactone	1711.50	1978.23	1.09	291.18±48.55	327.16±75.19	340.30±26.10	332.11±41.67	303.05±19.18
1-Octanol	1653.10	1743.78	1.49	372.66±80.84[ab]	453.50±26.17[a]	348.23±8.80[b]	421.30±61.44[ab]	413.89±32.20[ab]
(E)-2-Nonenal	1575.70	1475.26	1.42	311.60±35.84[b]	433.12±23.02[a]	315.57±10.82[b]	345.23±16.98[b]	303.44±21.26[b]
Benzaldehyde-M	1554.00	1407.57	1.16	5074.39±125.60[bc]	5405.43±240.70[a]	5237.33±92.26[ab]	4834.66±174.62[c]	4796.24±94.77[c]
Benzaldehyde-D	1554.00	1407.57	1.47	708.76±51.68[a]	797.58±91.43[a]	723.84±41.61[a]	590.70±35.12[b]	578.62±21.30[b]
1-Octen-3-ol	1489.70	1225.18	1.17	774.07±13.22[c]	1095.07±22.68[a]	595.86±7.57[d]	855.06±42.70[b]	638.60±56.31[d]
Methional	1479.40	1197.98	1.10	942.22±26.38[bc]	990.56±88.44[b]	1260.15±63.43[a]	850.69±85.92[c]	1139.01±63.62[a]
Tetramethylpyrazine	1479.40	1197.98	1.22	389.67±16.82[b]	423.49±43.53[b]	591.96±18.04[a]	394.61±50.26[b]	569.22±38.01[a]
2-Ethyl-1-hexanol	1490.20	1226.41	1.44	176.19±15.85[c]	259.31±6.36[a]	166.58±9.51[c]	236.85±12.36[b]	187.00±13.54[c]
(E)-2-Octenal	1442.20	1105.57	1.34	621.07±27.88[c]	1084.59±22.06[a]	604.71±23.21[c]	728.25±50.30[b]	645.17±27.62[c]
2-Ethyl-3-methylpyrazine	1433.70	1085.43	1.17	324.12±35.70[ab]	367.64±43.75[a]	337.90±2.69[ab]	313.23±20.72[ab]	294.27±27.85[b]
Dipropyl disulfide	1421.90	1058.18	1.27	159.09±9.00[c]	334.45±7.90[a]	146.68±14.99[c]	283.09±25.77[b]	171.51±38.63[c]
Nonanal-M	1407.70	1026.20	1.48	1254.62±96.83[b]	1603.05±24.36[a]	1147.67±8.90[c]	1309.54±70.52[b]	1131.83±32.44[c]
Nonanal-D	1407.70	1026.20	1.94	86.29±22.30[b]	140.77±5.33[a]	74.61±2.60[bc]	86.59±12.10[b]	62.96±3.67[c]
1-Hexanol	1373.40	952.75	1.33	348.95±2.69[b]	407.90±17.08[a]	263.87±7.09[c]	263.67±11.38[c]	218.74±11.98[d]
Hexyl propionate	1345.50	897.06	1.44	437.29±14.09[b]	898.84±60.44[a]	422.35±23.72[bc]	437.53±17.99[b]	366.84±38.84[c]
(E)-2-Heptenal	1335.00	877.01	1.26	204.08±8.64[c]	479.74±14.53[a]	191.93±22.11[c]	345.30±57.75[b]	291.85±47.99[b]
Propyl hexanoate	1335.60	878.10	1.38	668.24±6.26[c]	1265.59±38.70[a]	659.69±83.48[c]	1040.01±98.37[b]	959.38±114.03[b]
2,5-Dimethylpyrazine	1317.60	844.54	1.10	292.02±6.00[c]	307.15±103.75[c]	520.40±7.63[b]	609.55±148.85[ab]	680.55±32.32[a]
Cyclohexanone	1303.10	818.56	1.16	737.19±26.92[b]	662.29±12.78[b]	729.24±3.59[b]	670.63±123.61[b]	862.46±9.77[a]

Isoamyl 3-methylbutyrate	1304.30	820.73	1.47	470.58±6.35^{b}	732.76±12.44^{a}	417.17±17.80^{c}	498.68±19.70^{b}	424.24±24.50^{c}
Octanal	1298.80	810.99	1.42	333.67±12.48^{c}	476.45±28.95^{a}	420.29±4.70^{b}	461.27±17.80^{a}	395.94±14.51^{b}
Methylpyrazine	1281.00	781.76	1.09	257.30±6.15^{c}	254.25±20.64^{c}	500.54±4.41^{a}	340.67±38.86^{b}	472.78±7.31^{a}
Hexyl acetate	1275.50	773.10	1.38	193.46±17.53^{b}	182.80±13.58^{bc}	274.76±15.44^{a}	140.42±3.75^{d}	165.89±4.79^{c}
Styrene	1267.90	761.20	1.03	321.54±10.83^{a}	298.27±17.98^{b}	328.09±2.70^{a}	261.41±12.20^{c}	241.76±1.90^{c}
1-Pentanol	1268.60	762.28	1.29	133.17±2.50^{c}	249.58±7.12^{a}	119.58±4.31^{c}	150.19±8.58^{b}	125.06±14.44^{c}
Ammonia-D	1229.50	703.86	0.85	72246.36±3147.70^{c}	69389.19±3672.54^{c}	81056.77±581.88^{ab}	78274.53±3588.68^{b}	84989.52±1683.83^{a}
Ammonia-M	1230.80	705.71	0.91	55163.01±414.23^{a}	52764.68±500.30^{b}	52185.13±195.50^{b}	52909.23±522.86^{b}	52247.61±529.5^{b}
1.00	1235.20	712.16	1.30	307.84±2.50^{c}	745.51±7.52^{a}	297.94±46.79^{c}	535.67±74.74^{b}	487.12±77.07^{b}
(E)-2-Hexenal	1219.10	689.12	1.20	574.11±12.44^{ab}	533.81±126.64^{b}	681.15±15.58^{a}	629.58±32.20^{ab}	684.27±30.71^{a}
2.00	1217.80	687.27	1.33	214.28±13.51^{a}	197.99±23.75^{a}	152.24±13.36^{b}	140.22±20.22^{b}	128.39±7.26^{b}
Heptanal-M	1201.70	665.16	1.34	1312.72±26.87^{a}	1272.04±17.86^{b}	1197.27±8.78^{c}	1233.95±18.78^{b}	1226.95±28.06^{c}
Heptanal-D	1201.70	665.16	1.69	1087.69±99.11^{b}	1387.61±64.11^{a}	806.83±41.18^{cd}	898.80±40.53^{c}	765.10±79.01^{d}
2-Heptanone-M	1194.90	655.94	1.26	711.87±12.52^{a}	654.15±29.66^{b}	642.52±6.90^{b}	642.61±15.29^{b}	591.60±20.87^{c}
2-Heptanone-D	1196.20	657.78	1.63	990.01±53.72^{b}	1192.22±54.36^{a}	983.03±10.52^{b}	964.49±51.59^{b}	803.74±14.07^{c}
Pyridine	1199.70	662.39	1.24	378.13±1.04^{b}	353.37±18.05^{b}	449.67±10.98^{a}	354.37±23.65^{b}	351.23±25.59^{b}
3.00	1230.10	704.78	1.05	103.05±3.69^{c}	92.24±2.23^{d}	153.16±1.83^{a}	119.17±10^{b}	145.82±2.93^{a}
Ethyl hexanoate	1252.10	737.04	1.33	37.07±1.26^{b}	62.73±3.41^{a}	34.57±2.90^{bc}	33.04±1.64^{bc}	31.16±0.16^{c}
1-Penten-3-ol	1179.00	623.68	0.94	830.77±46.75^{c}	1190.50±11.24^{a}	747.08±42.72^{c}	1007.93±23.33^{b}	840.94±139.93^{c}
Myrcene	1178.60	622.76	1.22	278.42±5.72^{c}	371.01±2.74^{a}	331.65±8.90^{b}	359.43±12.81^{a}	332.37±14.61^{b}
Ethyl pentanoate	1175.50	616.31	1.27	208.44±6.20^{b}	349.33±31.80^{a}	202.84±11.28^{b}	193.09±13.59^{b}	151.33±13.05^{c}
1-Butanol-M	1163.50	591.43	1.18	974.13±46.15^{a}	1033.78±32.59^{a}	792.08±55.96^{b}	733.96±44.07^{bc}	689.89±31.76^{c}
1-Butanol-D	1163.90	592.35	1.38	183.77±12.76^{b}	256.66±19.69^{a}	131.66±18.48^{c}	123.36±14.91^{c}	115.69±10.30^{c}
(E)-2-Pentenal	1153.20	570.95	1.11	346.82±2.68^{c}	529.07±10.95^{a}	269.23±24.91^{d}	442.94±13.01^{b}	338.88±54.64^{c}

4.00	1149.00	562.69	1.08	380.08±30.09^{b}	348.78±11.40^{c}	433.33±9.00^{a}	363.34±2.93^{bc}	438.06±2.93^{a}
3-Carene	1150.40	565.45	1.22	715.25±17.60^{a}	628.52±26.69^{bc}	652.42±9.96^{b}	586.28±33.36^{c}	523.55±28.75^{d}
2,3-Heptanedione	1149.50	563.79	1.33	200.00±13.77^{c}	248.42±21.00^{b}	277.87±13.68^{a}	173.90±1.98^{d}	210.65±6.22^{c}
5.00	1149.80	564.35	1.37	303.98±17.11^{b}	349.77±29.39^{a}	292.54±12.59^{b}	212.64±8.22^{c}	201.18±9.41c
Isoamyl acetate	1143.50	552.23	1.30	312.92±47.83^{ab}	347.12±39.68^{a}	261.80±8.06^{bc}	313.94±14.37^{ab}	250.39±33.16^{c}
beta-Pinene	1136.40	539.02	1.21	603.08±97.94^{a}	612.04±39.45^{a}	479.64±22.29^{b}	356.82±29.55^{c}	372.82±19.52^{c}
(Z)-2-Pentenal-M	1119.20	508.20	1.10	1104.78±65.76^{a}	733.24±84.35^{d}	1048.49±18.41^{ab}	903.30±58.34^{c}	952.74±28.15^{bc}
(Z)-2-Pentenal-D	1119.90	509.30	1.35	1270.15±76.74^{b}	1235.41±110.34^{b}	1399.51±51.06^{a}	990.52±29.94^{c}	1049.79±11.62^{c}
Ethylbenzene	1135.50	537.37	1.09	99.14±8.24^{b}	74.17±3.09^{c}	114.11±1.56^{a}	85.70±10.01^{c}	110.85±6.68^{ab}
2-Methyl-1-propanol-M	1113.50	498.29	1.17	570.31±34.93^{a}	285.41±61.08^{d}	546.87±13.94^{ab}	455.48±88.36^{bc}	431.80±27.71^{c}
2-Methyl-1-propanol-D	1113.50	498.29	1.37	185.44±1.60^{a}	99.92±14.87^{bc}	134.39±1.69^{b}	126.37±40.67^{b}	80.48±10.53^{c}
Hexanal-M	1105.00	483.98	1.28	2165.17±92.08	1988.67±379.33	2123.28±13.59	2104.64±397.68	2006.33±56.09
Hexanal-D	1105.30	484.53	1.56	6975.18±21.51^{b}	7777.69±211.37^{a}	6065.69±199.64^{c}	6500.52±385.86^{bc}	6258.47±356.15^{c}
Camphene	1069.00	434.43	1.20	2330.92±134.48^{a}	2116.47±171.63^{b}	2454.16±83.06^{a}	1637.23±42.66^{c}	1761.78±41.12^{c}
1-Propanol	1058.80	421.77	1.11	854.13±13.13^{b}	930.74±23.35^{a}	667.42±10.51^{d}	718.36±24.61^{c}	670.14±4.96^{d}
1-Penten-3-one	1045.90	406.36	1.08	111.99±2.15^{b}	126.43±7.09^{a}	87.57±0.86^{c}	133.73±7.20^{a}	104.52±7.19^{b}
2-Butanol	1043.10	403.06	1.15	385.29±22.89^{a}	366.61±4.61^{a}	376.93±11.14^{a}	327.66±33.16^{b}	322.33±7.09^{b}
Isobutyl acetate	1035.90	394.80	1.23	1017.06±13.24^{b}	924.45±29.47^{bc}	1133.89±37.10^{a}	725.79±90.19^{d}	864.09±57.29^{c}
6.00	1030.00	388.19	1.14	202.19±2.12^{a}	171.82±2.97^{b}	132.10±6.16^{d}	143.49±5.59^{c}	113.75±3.69^{e}
Methyl 2-methylbutanoate	1016.50	373.33	1.51	2540.48±252.85^{abc}	2749.44±196.48^{a}	2352.98±68.20^{bc}	2637.75±122.70^{ab}	2235.07±136.05^{c}
Pentanal	1007.70	363.97	1.42	5267.22±26.25^{b}	5686.42±100.63^{a}	4826.02±82.12^{c}	5296.64±27^{b}	4933.99±174.82^{c}
2-Pentanone	1002.90	359.02	1.36	1047.28±47.57^{bc}	1160.41±6.42^{a}	1024.48±8.56^{c}	1079.76±28.61^{bc}	1099.33±42.62^{b}
2-Ethylfuran	975.40	336.45	1.04	1133.17±36.08^{b}	1485.20±56.02^{a}	828.77±21.51^{d}	1013.73±9.73^{c}	918.60±85.89^{d}
4-Methyl-2-pentanone	1030.50	388.74	1.48	182.54±16.95^{b}	158.92±15.91^{c}	214.35±10.07^{a}	136.22±7.81^{c}	144.56±6.19^{c}

2,5-Dimethylfuran	951.60	319.38	1.37	3453.13±168.19^{b}	3853.30±195.62^{a}	3720.25±102.89^{ab}	3801.19±169.69^{a}	3651.84±91.81^{ab}
3-Methylbutanal	931.40	305.62	1.40	3520.51±42.75^{a}	3442.10±78.17^{ab}	3277.38±22.18^{c}	3372.13±66.24^{bc}	3097.01±66.34^{d}
7.00	984.30	343.05	1.41	771.60±66.53^{c}	729.25±39.20^{c}	1181.79±21.86^{a}	779.94±15.75^{c}	874.44±35.37^{b}
8.00	964.90	328.82	1.41	710.78±58.61^{c}	677.32±21.15^{c}	954.83±16.50^{a}	810.51±22.72^{b}	817.93±20.65^{b}
2-Butanone	918.90	297.39	1.25	7299.37±49.36^{a}	7163.66±113.47^{bc}	7264.02±11.72^{ab}	7117.89±36.89^{c}	7317.40±32.9^{a}
Ethyl acetate	901.60	286.35	1.34	1924.25±38.09^{a}	1487.20±118.30^{b}	1405.81±119.29^{b}	1387.45±116.23^{b}	1156.90±51.35^{c}
Butanal	893.50	281.34	1.28	1366.91±39.98^{b}	1563.51±54.63^{a}	1158.27±20.20^{c}	1222.63±19.09^{c}	1026.93±54.59^{d}
Acetone	845.30	253.25	1.11	11034.67±226.07^{c}	11244.86±283.99^{bc}	11478.79±80.83^{ab}	11241.21±9.08^{bc}	11737.02±128.58^{a}
2-Methylpropanal	831.20	245.56	1.28	583.05±61.84^{a}	450.76±13.21^{b}	546.97±6.81^{a}	545.06±2.33^{a}	533.34±13.50^{a}
Propanal	821.70	240.54	1.14	3873.54±44.01^{b}	4325.42±50.34^{a}	3851.84±35.51^{b}	3883.59±73.66^{b}	3607.63±104.46^{c}
Dimethyl sulfide	796.20	227.50	1.09	4912.46±83.28^{a}	4508.16±145.80^{b}	4330.37±8.83^{c}	3894.49±42.06^{e}	4139.86±16.25^{d}
Acetaldehyde	768.50	214.13	1.02	822.92±39.13^{a}	666.52±80.37^{bc}	723.47±11.95^{b}	560.38±52.34^{d}	594.36±34.60^{cd}
2-Propenal	870.00	267.29	1.06	343.23±12.04^{b}	411.38±28.64^{a}	294.98±20.14^{c}	379.57±25.26^{ab}	291.35±12.58^{c}
9.00	892.90	281.00	1.20	592.49±8.52^{c}	671.03±4.40^{a}	623.79±7.77^{b}	639.86±8.96^{b}	638.05±26.13^{b}
1-Propanethiol	841.70	251.24	1.36	348.72±3.77^{d}	364.67±33.20^{cd}	548.62±15.09^{a}	383.22±7.07^{c}	501.75±15.05^{b}
Diacetyl	973.20	334.84	1.18	436.25±18.15^{c}	484.93±11.27^{b}	600.35±23.92^{a}	526.26±34.41^{b}	571.78±27.48^{a}
p-Xylene	1126.60	521.15	1.05	156.54±6.20^{c}	106.00±5.18^{d}	234.14±1.48^{a}	174.66±21.14^{c}	210.06±1.33^{b}
Furfural	1500.40	1253.79	1.09	130.19±8.82^{b}	148.09±17.24^{b}	201.17±4.29^{a}	142.13±14.51^{b}	195.59±11.26^{a}
2-Heptanol	1315.50	840.77	1.39	194.11±2.32^{b}	309.90±13.41^{a}	221.02±4.06^{b}	218.66±29.07^{b}	201.82±16.48^{b}
1-Hydroxy-2-propanone	1318.60	846.50	1.06	79.27±3.88^{d}	101.18±15.06^{cd}	117.61±1.36^{bc}	145.53±26.80^{a}	135.47±7.42^{ab}
Acetoin	1302.30	817.11	1.06	118.61±3.28^{ab}	123.11±5.90^{a}	109.22±4.15^{b}	106.56±11.4^{4b}	108.57±6.75^{b}
2-Ethylpyrazine	1331.50	870.37	1.12	56.12±1.76^{e}	85.33±0.98^{c}	117.13±2.10^{a}	73.20±5.42^{d}	95.47±3.65^{b}
2,6-Dimethylpyrazine	1337.20	881.18	1.14	34.98±2.31^{a}	51.49±2.99^{a}	50.17±1.07^{a}	42.25±2.16^{b}	41.45±1.36^{b}
(Z)-4-Heptenal	1237.60	715.55	1.15	52.89±2.18^{d}	77.57±6.34^{ab}	81.82±5.13^{a}	71.94±2.49^{bc}	66.91±6.79^{c}

Propanoic acid	1665.50	1791.14	1.10	215.71±37.89^{b}	239.02±22.79^{b}	289.92±9.98^{a}	245.01±9.31^{b}	254.27±9.34^{ab}
Decanal	1529.60	1335.33	1.55	233.53±20.30^{b}	288.46±43.38^{a}	259.40±21.92^{ab}	286.48±27.45^{a}	253.64±2.75^{ab}
3-Methyl-1-butanol	1214.90	683.21	1.25	269.44±11.23^{a}	154.86±51.09^{bc}	174.57±9.26^{b}	148.76±21.20^{bc}	116.39±13.65^{c}
3-Pentanol	1112.20	496.15	1.43	312.60±53.06^{a}	232.42±62.49^{b}	240.06±16.75^{b}	202.80±22.17^{bc}	146.93±15.79^{c}
2-Hexanone	1104.60	483.34	1.49	361.75±2.64^{b}	389.32±35.42^{b}	488.23±9.45^{a}	386.92±22.46^{b}	388.64±8.78^{b}

The data are presented as the mean±SD; Different letters within the same row indicate significant differences ($p < 0.05$).
